# Impact of a fermented soy beverage supplemented with acerola by-product on the gut microbiota from lean and obese subjects using an *in vitro* model of the human colon

**DOI:** 10.1007/s00253-021-11252-8

**Published:** 2021-05-03

**Authors:** Antonio Diogo Silva Vieira, Carlota Bussolo de Souza, Marina Padilha, Erwin Gerard Zoetendal, Hauke Smidt, Susana Marta Isay Saad, Koen Venema

**Affiliations:** 1 Department of Biochemical and Pharmaceutical Technology, School of Pharmaceutical Sciences, University of São Paulo (USP), Av. Professor Lineu Prestes, 580, São Paulo, SP, 05508-000 Brazil; 2 Food Research Center FoRC, University of São Paulo (USP), Av. Professor Lineu Prestes, 580, São Paulo, SP, 05508-000 Brazil; 3 Centre for Healthy Eating & Food Innovation, Maastricht University - Campus Venlo, Villafloraweg 1, 5928 SZ, Venlo, The Netherlands; 4 Laboratory of Microbiology, Wageningen University & Research, Stippeneng 4, 6708 WE Wageningen, The Netherlands

**Keywords:** Probiotic, Fruit by-product, Gut microbiota, Obesity, *In vitro* model

## Abstract

**Abstract:**

The aim of this study was to evaluate the effects of soy-based beverages manufactured with water-soluble soy extract, containing probiotic strains (*Lactobacillus acidophilus* LA-5 and *Bifidobacterium longum* BB-46) and/or acerola by-product (ABP) on pooled faecal microbiota obtained from lean and obese donors. Four fermented soy beverages (FSs) (“placebo” (FS-Pla), probiotic (FS-Pro), prebiotic (FS-Pre), and synbiotic (FS-Syn)) were subjected to *in vitro* digestion, followed by inoculation in the TIM-2 system, a dynamic *in vitro* model that mimics the conditions of the human colon. Short- and branched-chain fatty acids (SCFA and BCFA) and microbiota composition were determined. Upon colonic fermentation in the presence of the different FSs formulations, acetic and lactic acid production was higher than the control treatment for faecal microbiota from lean individuals (FMLI). Additionally, SCFA production by the FMLI was higher than for the faecal microbiota from obese individuals (FMOI). *Bifidobacterium* spp. and *Lactobacillus* spp. populations increased during simulated colonic fermentation in the presence of FS-Syn in the FMLI and FMOI. FS formulations also changed the composition of the FMOI, resulting in a profile more similar to the FMLI. The changes in the composition and the increase in SCFA production observed for the FMLI and FMOI during these *in vitro* fermentations suggest a potential modulation effect of these microbiotas by the consumption of functional FSs.

**Graphical abstract:**

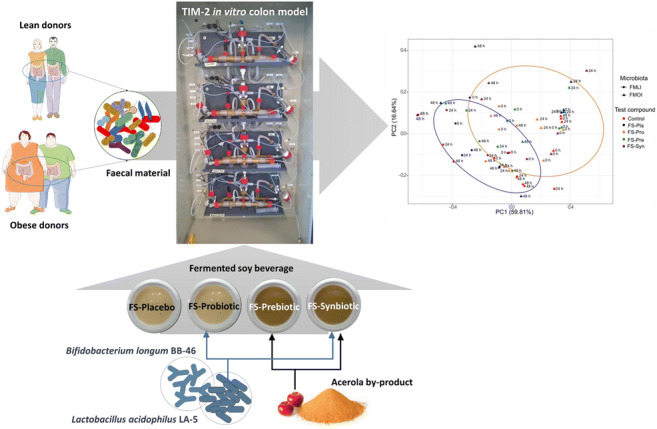

**Key points:**

• *Soy beverages increased Bifidobacterium abundance in microbiota from obese individuals.*

• *The synbiotic beverage increased Bifidobacterium abundance in microbiota from lean individuals.*

• *The synbiotic beverage changed the microbiota from obese individuals, approaching the lean profiles.*

**Supplementary Information:**

The online version contains supplementary material available at 10.1007/s00253-021-11252-8.

## Introduction

Fermented functional food, especially dairy products containing probiotic bacteria, “live microorganisms that, when administrated in adequate amounts, confer a health benefit on the host” (Hill et al. 2014), and prebiotic ingredients, “substrate that is selectively utilized by host microorganisms conferring a health benefit” (Gibson et al. 2017), have been extensively explored by researchers and the food industry (Vinderola et al. 2017). However, given the growth of vegetarianism, a high incidence of lactose intolerance, and allergy to milk proteins, besides the high cholesterol content in dairy products, the replacement of milk by water-soluble soy extract has been a promising alternative in the development of products (O’Toole 2016; Vinderola et al. 2017). Several health benefits are associated to the consumption of soy-based foods, for example, the reduction of cardiovascular disease risk (Bedani et al. 2015; Padhi et al. 2016), immunomodulatory activity (Lin et al. 2016), and decreased formation of putrefactive compound by the gut microbiota (Nakata et al. 2017).

Acerola (*Malpighia emarginata* DC) is a fruit known for its high content of vitamin C, phenolic compounds, anthocyanins, and carotenoids (Belwal et al. 2018). There is an emerging interest in acerola as a nutraceutical or functional food with remarkable market value, especially related to its extracts and bioactive compounds. These compounds have been related to health benefits including antioxidant and antitumor activity, antihyperglycemic effect, and skin protection activity, besides an increase in the adhesion of probiotic strains in the Caco-2 cell line model (Albuquerque et al. 2019; Belwal et al. 2018). Additionally, the acerola by-product (ABP), which is the material remaining after obtaining acerola juice, showed a high content of total dietary fibre, representing 48.46% to 56.28% of its dry matter (Bianchi et al. 2019; Vieira et al. 2017). ABP fermentation was reported to stimulate the relative abundance of *Lactobacillus* and *Bifidobacterium* (Bianchi et al. 2019). This selective use of ABP as a source of fermentable compounds by beneficial microorganisms but not by members of the *Clostridia* class and *Escherichia coli* is one of the first characteristics expected from an ingredient with a potential prebiotic effect (Gibson et al. 2017).

Some strains of *Bifidobacterium longum*, which is one of the most abundant species in the healthy human gut microbiota, have been used as probiotics, and benefits to health and wellness have been widely reported (Zhang et al. 2019). An anti-obesity effect was observed in rats after supplementation for 15 days with a *B. longum* strain alone or in combination with *Lactobacillus casei* Shirota, leading to a significant reduction in body weight and serum triglycerides in the high-fat diet (HFD)–fed rats (Karimi et al. 2017). *Lactobacillus acidophilus* LA-5 is one of the most studied probiotics. No evidence of the direct effects of this strain on obesity has been reported. However, studies are demonstrating that this probiotic has beneficial effects on several diseases and syndromes associated with obesity. These effects include anti-diabetic and anti-inflammatory effects in patients with type 2 diabetes mellitus (T2DM) (Ejtahed et al. 2012), reduction of inflammatory mediators in obese and overweight people (Zarrati et al. 2013), a decrease in LDL-cholesterol (LDL-C) and in LDL-C to HDL-C ratio in normocholesterolemic men (Bedani et al. 2015), and a decrease in the level of blood glucose and markers of vascular cell adhesion molecule cell (VCAM-1) in people with metabolic syndrome (Rezazadeh et al. 2019).

A food product should only be considered a probiotic product when containing live microorganisms with a suitable viable count of well-defined strains. Preserving the probiotic viability in the product has been a prerequisite for ensuring its effect on the health of the host (Hill et al. 2014; Wan et al. 2019). Among the mechanisms to promote health benefits, modulation of the gut microbiota, with the production of organic acids, especially short-chain fatty acids (SCFA); competitive exclusion of pathogens; and regulation of the intestinal transit have been frequently studied (Hill et al. 2014). Modulation of the intestinal microbiota by dietary intervention, with the inclusion of probiotics and prebiotics, alone or in combination (synbiotic), might be a promising alternative to prevent and treat obesity as well as related diseases such as metabolic syndrome, cardiovascular disease, and T2DM (Canfora et al. 2019; Reid et al. 2017; Torres-Fuentes et al. 2017).

The fermented soy beverages (FSs) used in this study were produced with water-soluble soy extract and probiotic microorganisms previously selected based on bile salt deconjugation ability (Vieira et al. 2019). They turned out to be a promising vehicle for probiotic strains *L. acidophilus* LA-5 and *B. longum* BB-46, which were viable (≥ 7.0 log colony forming units (CFU) equivalent/mL) during 28 days at 4 °C (Vieira et al. 2021). The FSs also appeared to be good sources of essential fatty acids (ω-9, ω-6, and ω-3) and to have good sensory acceptance (Vieira et al. 2019). Moreover, FS products were supplemented with ABP, which contributed to approximately 1% of dietary fibre in the FS chemical composition and increased *B. longum* BB-46 survival under simulation of *in vitro* gastrointestinal conditions, when in co-culture with *L. acidophilus* LA-5 (Vieira et al. 2019; 2021). In this way, the perspective of using a fermented soy-based product (100% from vegetables) as food matrix with potential for advantageous changes in the intestinal microbiota of lean and obese individuals seemed to be promising. Therefore, in order to evaluate the possible benefits that the consumption of FSs may have, the aim of this study was to evaluate the impact of probiotic-containing FSs supplemented with acerola by-product on the composition and metabolic activity of human lean and obese microbiota, using the TIM-2 *in vitro* colon system (Minekus et al. 1999).

## Material and methods

### Probiotic and starter cultures and acerola by-product origin

Probiotic (*L. acidophilus* LA-5 and *B. longum* BB-46) and starter (*Streptococcus thermophilus* TH-4) cultures were supplied by Chr. Hansen (Hørsholm, Denmark). Before inoculation into the pasteurized soy-based mixtures, the stock cultures stored at − 80 °C were activated with two successive transfers at 37 °C for 24 h, under oxic conditions for the *L. acidophilus* LA-5 and *S. thermophilus* TH-4, and under anoxic conditions for *B. longum* BB-46 according to a study by Vieira et al. (2019). After the second activation, 250 mL of the inoculum of each culture containing approximately 8.0 ± 0.4 log CFU/mL was washed twice with sterile NaCl solution (0.85%, w/v). The pellet was collected by centrifugation (10,000 *g* for 10 min at 4 °C, K243R, Eppendorf, Hamburg, Germany) and stored at 4 °C for not more than 1 h. The origin of the ABP (*M. emarginata* DC) powder (seeds and peels) and its preparation were previously described in Vieira et al. (2017). The ABP powder was obtained by drying in an air flow oven at 60 °C for 24 h, followed by trituration to obtain a fine powder less than 0.42 mm of diameter. The powder was stored at − 18 °C in vacuum-sealed plastic bags in portions of 200 g.

### Fermented soy beverage experimental design and production

Four different formulations of FSs were produced, employing a randomized 2^2^ factorial design, in duplicate for each fermentation assay by the faecal microbiota studied, in order to evaluate the presence or absence of both the probiotic combination (*B. longum* BB-46 and *L. acidophilus* LA-5) and the ABP (Table 1). The FSs were adapted to be produced in The Netherlands, following the same procedures and formulations previously developed in Brazil (Vieira et al. 2019), as follows: water-soluble soy extract powder (200 g/L, Mãe Terra, São Paulo, Brazil) was diluted in distilled water, employing a hand blender (Bosch, Skofja Loka, Slovenia) and heated in an electric stove under constant agitation. After reaching 50 °C, sucrose (50 g/L, Jumbo Supermarkten B.V., Veghel, The Netherlands) and dextrose (10 g/L, Roquette, Lestrem, France) were added and mixed with a hand blender for approximately 1 min. Heating continued until reaching 80 °C, when carrageen gum (1 g/L, ETM 3, AgarGel, São Paulo, Brazil) was added and mixed again until the complete dissolution of the gum. When the mixture reached 90 °C, it was pasteurized (90 °C for 5 min). For formulations FS-Pre and FS-Syn, when the mixture reached 90 °C, the ABP powder (20 g/L) was added, and the mixture was pasteurized (90 °C for 5 min). Next, all soy-based mixtures were cooled in an ice bath to 37 °C, for the addition of inoculum (*S. thermophilus* and/or probiotics), followed by incubation at 37 °C in a water-bath (Julabo®, Seelbach, Germany) until reaching pH 5.5. Afterwards, the FSs were cooled and kept at 4 °C for 2 h, when concentrated acerola juice (100 g/kg, Acerola Jal, Citro-Nutri, Olaria, RJ, Brazil) was added and mixed. Next, the FSs were packaged in plastic containers and stored at 4 °C. All FSs were produced in batches of 1 L.

**Table 1:** Variables employed in the production of the fermented soy beverages studied

Fermented soy beverage (FS)	Factors studied	
	Probiotic combination *L. acidophilus* LA-5 *+ B. longum* BB-46	Acerola by-product
Placebo-FS-Pla	-	-
Probiotic-FS-Pro	+	-
Prebiotic-FS-Pre	-	+
Synbiotic-FS-Syn	+	+

+ = presence, - =absence

### Lean and obese faeces collection and standardization

Faecal samples were obtained from recruited healthy volunteers. The faeces from lean individuals were obtained from five volunteers (two males, three females) aged between 20 and 33 years and with an average body mass index (BMI) of 21.69 kg/m^2^ ± 0.90. The faeces from obese individuals were obtained from thirteen volunteers (six males, seven females) aged between 31 and 67 years and with an average BMI of 33.20 kg/m^2^ ± 3.70. Before donating their faeces, volunteers signed an informed consent form. The volunteers were all non-smokers who had not used probiotics, prebiotics, antibiotics or laxatives for the 3 months preceding donation. The fresh faecal samples were collected in a gastight bag and stored in a plastic jar containing an anaerobiosis generator (AnaeroGen™, Oxoid™, Basingstoke, UK), which was transported in a cool box with ice to the laboratory in less than 5 h. After arrival, they were homogenized and mixed in an anaerobic chamber with a dialysate solution (pH set to 5.8) formulated as described previously by Cuevas-Tena et al. (2019) (Table S1 of Supplementary information) and 140 g/L of glycerol (Aguirre et al. 2014b; 2015). Afterwards, the samples were fractionated into portions of 30 mL in centrifuge tubes of 50 mL, followed by snap-freezing in liquid nitrogen and storage at − 80 °C until the experiments in the TIM-2 system proceeded (Aguirre et al. 2015).

### Pre-digestion of the fermented soy beverages

The FSs were submitted to a pre-digestion in 3 steps (gastric phase, pH set 2.0–2.2; enteric phase I, pH set 4.5–4.7; and enteric II phase, pH set 5.5–5.9) (Buriti et al. 2010) after 6 and 7 days of storage (estimated time for shipping and selling of fermented products). For each step of pre-digestion, the FSs were incubated in a water bath (Julabo®) in the presence of the gastric or enteric simulated juices described by Buriti et al. (2010) at 37 °C for 2 h under constant agitation of 150 rpm (details in Supplementary information Text S1). The same condition used for the FSs pre-digestion was also carried out on 25 g of dialysate solution, which was introduced in TIM-2 as an additional control, which excluded the food matrix of the fermented soy beverage (FS). In total, 6 h of pre-digestion resulted in a volume of 52.5 mL to be introduced into the TIM-2 system. The survival of probiotic and starter strains after pre-digestion were determined by PMA-qPCR, as described below. Pre-digested solutions were introduced into a TIM-2 unit as a single shot, with a mean amount of surviving cells of *S. thermophilus* TH-4 of 10.19 ± 0.94 log cells/shot for all FS. For the beverages FS-Pro and FS-Syn, the *L. acidophilus *LA-5 and *B. longum *BB-46 mean survival cell counts were 7.25 ± 1.04 log cells/shot and 9.30 ± 0.64 log cells/shot, respectively.

### TIM-2 experimental protocol

**Fig. 1:**
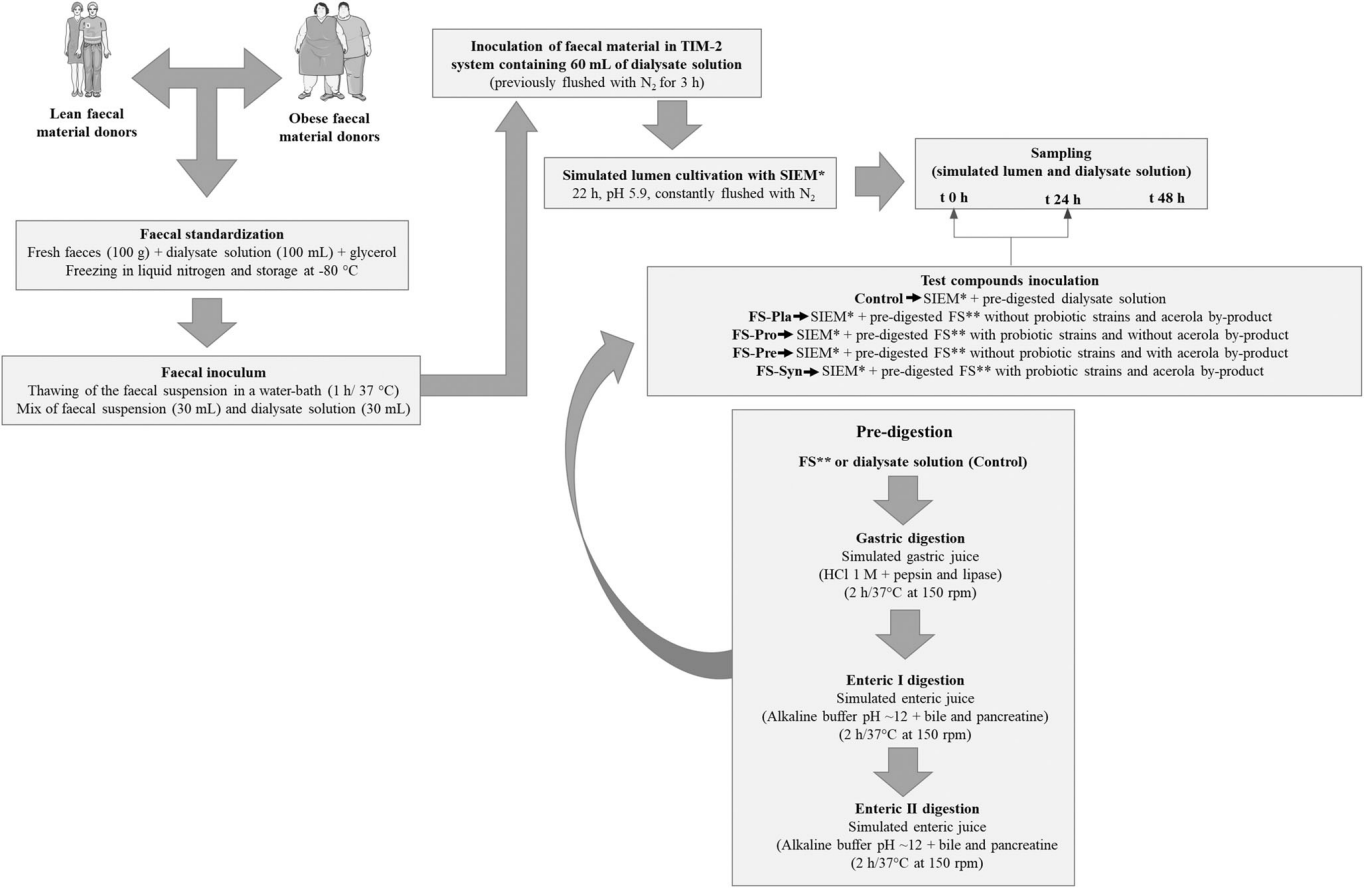
Schematic representation of the experimental set up with timeline for the TIM-2 model. *Standard Ileal Efflux Media (SIEM). **Fermented soy beverage (FS)

The experiments were performed in duplicate (*n* = 2) for each of the FS and the control, both for faecal microbiota from lean individuals (FMLI) and faecal microbiota from obese individuals (FMOI), with a total of 20 independent fermentation assays (5 test compounds × 2 microbiotas × 2 replicates). The TIM-2 system consisting of four independent units that can be run in parallel (Fig. S1, Supplementary information) was described in detail by Aguirre et al. (2014a) and Minekus et al. (1999). Before each experiment, TIM-2 units containing 60 mL of dialysate solution (Table S1, Supplementary information) were flushed for 3 h with N_2_ prior to the introduction of the microbiota inoculum. The microbiota inoculum containing 30 mL of standardized faecal samples was thawed at 37 °C for 1 h in a water bath and homogenized with 30 mL of pre-reduced dialysate solution in an anaerobic chamber (Fig. 1) (Aguirre et al. 2015). The simulated lumen was maintained at 37 °C for the entire period with the pH kept at or above 5.9 by automatic titration with 2 M NaOH, and the anoxic condition was kept by continued flushing of the system with N_2_ gas. A dialysate system (Supplementary information, Fig. S1) was responsible for removing excess volume and fermentation metabolites from the TIM-2 units. The microbiota was cultivated for 22 h with Standard Ileal Efflux Media (SIEM) composed of the average non-digestible carbohydrates consumed in a normal western diet (Cuevas-Tena et al. 2019; Gibson et al. 1988) (Supplementary information, Table S2). After this 22 h cultivation (adaptation period), a 48 h experimental period was started (Fig. 1). SIEM was added to all TIM-2 units throughout this period. Moreover, two shots of 52.5 mL of pre-digested FS or pre-digested dialysate solution (as the control) were fed to the microbiota daily (Fig. 1): immediately after the adaptation period (0 h) and after 24 h of fermentation, in each TIM-2 system (Fig. 1). To simulate the passage of material from the proximal to the distal colon and for the collection of samples for the microbiota and metabolic determinations, simulated lumen samples of a total of 25 mL were removed from the system, at 0 h (after the adaptation period), 24 h, and 48 h after the start of the experimental period (Fig. 1).

### Short-chain fatty acids, branched-chain fatty acids, and secondary organic acid determination

An aliquot of 1.5 mL of simulated lumen or 2 mL of spent dialysates from TIM-2, for each test compound sampled at 0, 24, and 48 h, was analyzed to determine microbial metabolites (SCFA; branched-chain fatty acids (BCFA); and lactic, formic, succinic, valeric, and caproic acids) (Cuevas-Tena et al. 2019). Samples were centrifuged (13,000 *g* for 10 min, at room temperature, K243R, Eppendorf), and the supernatant was filtered through a 0.45-μm PTFE filter followed by dilution with a mobile phase (1.5 mM aqueous sulphuric acid solution) in the proportions 1:5 and 1:2, for the simulated lumen and spent dialysates, respectively. Next, 10 μL of this mixture were loaded into the chromatograph through an automatic sampler 730 (Metrohm, Herisau, Switzerland). For the quantification of organic acids, ion exclusion chromatography (IEC) was used, employing an 883 chromatograph (IC, Metrohm) equipped with a Transgenomic IC Sep ICE-ION-300 column (30 cm × 7.8 mm × 7 μm) and a MetroSep RP2 Guard. The analysis of organic acids was performed by Brightlabs (Venlo, The Netherlands).

### Propidium monoazide treatment and DNA extractions from TIM-2 samples for qPCR

Aliquots of 200 μL of simulated lumen samples collected from TIM-2 units at 0, 24, and 48 h, as well as an aliquot of 500 μL of the pre-digested FS after 6 h of *in vitro* digestion, were washed twice with 500 μL of sterilized Tris-EDTA buffer (10 mM Tris-HCl, 1 mM EDTA, TE buffer, pH 8.0) and centrifuged at 15,700 *g* for 10 min at 4 °C (K243R, Eppendorf), followed by storage at − 20 °C, until the propidium monoazide (PMA) (phenanthridium, 3-amino-8-azido-5-[3-(diethylmethylammonio) propyl]-6-phenyl dichloride; Biotium, Inc., Hayward, CA, USA) treatment and DNA extraction took place. The PMA treatment followed the procedure described by Fujimoto et al. (2011), with slight modifications as described by Villarreal et al. (2013). DNA extraction from PMA-treated TIM-2 samples was performed according to Van Lingen et al. (2017). For DNA purification, the MaxWell® 16 Tissue Lev Total RNA purification kit (XAS1220, Promega, Madison, WI, USA) was employed, and the purified DNA was eluted in 50 μL of nuclease-free water (Qiagen, Germantown, MD, USA) (Van Lingen et al. 2017). DNA concentration and quality were determined using a DS-11 Microvolume Spectrophotometer (DS11SX, DeNovix, GL Biotech, Cambridge, UK).

### Quantitative real-time PCR

The reactions were conducted using an ABI-PRISM 7500 sequencing detection system (Applied Biosystems, Bridgewater, NJ, USA). The reaction mixtures (25 μL) contained the PCR Master Mix, with each primer at the adequate concentration (Table S3, Supplementary information), and 5 μL of the template DNA. For quantification of *Lactobacillus*, the amplification programme was 50 °C for 2 min, 95 °C for 10 min, and 40 cycles of 95 °C for 15 s, 58 °C for 20 s, and 72 °C for 30 s, adapted from Rinttilä et al. (2004). Quantification of total bacteria and *Bifidobacterium *was conducted according to Furet et al. (2009). For quantification of target species *L. acidophilus* and *S. thermophilus*, amplification programmes used were as described previously by Tabasco et al. (2007) and Falentin et al. (2012), respectively. For *B. longum*, the amplification programme was adapted from Gueimonde et al. (2007) and was as follows: 50 °C for 2 min, 95 °C for 10 min, and 40 cycles of 95 °C for 15 s, 65 °C for 1 min and 30 s. To distinguish the target from the non-target PCR products in Power SYBR Green quantitative real-time PCR (qPCR) reactions, the melting curve analysis followed amplification. In order to quantify each target microorganism and/or group, standard curves were generated by serially 10-fold dilutions of genomic DNA and/or 16S rRNA gene (10^8^–10^0^ copies per μL, except for total bacteria, which was 10^9^–10^1^ copies per μL) amplified from the respective target strains. For *L. acidophilus*, 16S rRNA was used, taking strain *L. acidophilus* NCFM as the reference strain, which contains four copies of the 16S rRNA gene in its genome (Altermann et al. 2005). Finally, the target count was estimated by matching the sample threshold cycle (*Ct*) with a standard curve *Ct*, in which coefficients of efficiency varied from 89 to 105% and the correlation coefficients (*r*^2^) from 0.99 to 1.00. Additionally, samples of non-template controls (NTC) were amplified in all qPCR runs and tested negative. Assays were all conducted in duplicate for TIM-2 trials and in triplicate for qPCR reactions (a total 6 individual values), and the means expressed as log cells/mL of simulated lumen were used for analysis.

### Microbiota profiling and bioinformatics

For the microbiota profiling of the simulated lumen samples from TIM-2, Illumina 16S rRNA gene amplicon libraries were generated and sequenced (Cuevas-Tena et al. 2019, Supplementary information Text S2). For the bioinformatic analysis of data, sequences were analyzed using the QIIME-pipeline version 1.9.1 (Caporaso et al. 2010). Unique sequences were aligned using the “align.seqs” command and an adaptation of the Bacterial SILVA SEED database as a template for taxonomic classification of Operational Taxonomic Unit (OTU) (Caporaso et al. 2010; Pruesse et al. 2007, Supplementary information Text S3). Alpha-diversity (Shannon Index, PD_whole_tree, Chao 1, and Observed_OTUs) and beta-diversity (distance matrices using unweighted and weighted UniFrac) measures were carried out (Caporaso et al. 2010). The results from alpha-diversity are shown in the Supplementary information (Figs. S2 and S3, and Table S4).

### Statistical analysis

The data of the amounts of total bacteria, *Bifidobacterium*, *Lactobacillus*, *L. acidophilus*, *B. longum*, and *S. thermophilus* from the PMA-qPCR analysis, as well as the cumulative content of SCFA, BCFA, and secondary organic acids, the *Prevotella/Bacteroides* ratios, and alpha-diversity, were submitted to non-parametric analyses of variance (ANOVA); the Mann-Whitney *U* test was applied in order to evaluate the microbiota effect and Kruskal-Wallis to evaluate the FS and sampling time effects, employing a significance level of *P* < 0.05. The Fisher LSD test was used for the comparison of means. The statistical package Statistica 13.0 (StatSoft, Tulsa, OK, USA) was employed, and the results were presented as means ± standard error (SE). We used Principal Coordinate Analysis (PCoA) to compare similarities between samples and tested differences using a Permutational Analysis of Variance (PERMANOVA) (Anderson 2001), employing a significance level of *P* < 0.05, using the statistical software R (R Development Core Team 2014) with *vegan* packages (Oksanen et al. 2013).

## Results

### Production of SCFA, BCFA, and secondary organic acids

Significant differences were observed between the FMLI and the FMOI for all SCFA, except for butyric acid (Table 2). Cumulative concentrations (after 48 h of fermentation in TIM-2) of acetic (*P* < 0.001) and lactic (*P* < 0.001) acid prevailed more in the FMLI. The cumulative concentrations of acetic, lactic, and formic acid were significantly higher for the synbiotic FS (FS-Syn) (116.56 mmol, 27.26 mmol, and 4.39 mmol, respectively) than for the control treatment (pre-digested dialysate) (72.65 mmol, 1.47 mmol, 2.68 mmol, respectively) for the FMLI (Table 2). In the FMOI, the cumulative concentrations of valeric and caproic acids were the highest for FS-Syn when compared to the control treatment. Acetic acid was the metabolite in the highest proportion in both the FMLI and the FMOI (Supplementary information, Fig. S4).

**Table 2:** Cumulative amount of SCFA, BCFA, and secondary organic acids (mmol) in the experiments with microbiota from lean and obese individuals for the different test compounds in the TIM-2 system (*n* = 2)

	Faecal microbiota from lean individuals (FMLI)					Faecal microbiota from obese individuals (FMOI)				
	Control	FS-Pla	FS-Pro	FS-Pre	FS-Syn	Control	FS-Pla	FS-Pro	FS-Pre	FS-Syn
Acetic acid										
24 h	41.62 (5.06)^Aa^	51.42 (10.17)^Aa^	57.27 (0.57)^Aa^	55.00 (2.97)^Aa^	58.42 (2.02)^Aa^	19.36 (1.89)^Ba^	20.10 (1.07)^Ba^	21.52 (0.79)^Ba^	12.52 (9.86)^Ba^	25.89 (9.33)^Aa^
48 h	72.65 (9.78)^Ab^	104.96 (1.31)^Aa^	104.89 (3.14)^Aa^	103.63 (1.86)^Aab^	116.56 (5.18)^Aa^	38.11 (1.89)^Ba^	38.89 (8.18)^Ba^	51.77 (4.80)^Ba^	41.73 (9.13)^Ba^	64.52 (2.96)^Ba^
Propionic acid										
24 h	7.30 (1.10)^Ba^	4.11 (1.46)^Bb^	5.35 (0.22)^Bab^	3.45 (0.94)^Bb^	6.08 (1.43)^Bab^	12.93 (0.40)^Aa^	14.19 (0.92)^Aa^	11.47 (0.03)^Aa^	14.29 (1.01)^Aa^	12.53 (0.99)^Aa^
48 h	21.69 (1.12)^Aa^	14.29 (2.73)^Aa^	17.31(2.38)^Aa^	12.29 (2.47)^Aa^	12.56 (1.36)^Ba^	25.92 (1.12)^Aa^	20.62 (1.71)^Aa^	21.66 (2.03)^Aa^	25.07 (1.68)^Aa^	28.88 (6.45)^Aa^
Butyric acid										
24 h	6.64 (0.14)^Aa^	7.85 (1.34)^Aa^	8.91 (0.14)^Aa^	9.96 (0.04)^Aa^	5.55 (0.03)^Aa^	8.10 (0.50)^Aa^	11.92 (1.69)^Aa^	11.20 (0.25)^Aa^	6.53 (5.62)^Aa^	12.10 (3.12)^Aa^
48 h	15.37 (0.05)^Aa^	21.37 (2.74)^Aa^	24.08 (0.57)^Aa^	25.65 (1.99)^Aa^	17.08 (5.87)^Aa^	14.67 (0.74)^Aa^	21.77 (6.09)^Aa^	23.12 (1.31)^Aa^	17.56 (4.86)^Aa^	26.41 (2.34)^Aa^
Valeric acid										
24 h	0.24 (0.00)^Ba^	0.23 (0.01)^Ba^	0.23 (0.00)^Ba^	0.24 (0.00)^Aa^	0.24 (0.00)^Ba^	2.02 (0.43)^Aab^	1.90 (0.25)^Aab^	3.20 (0.28)^Aa^	1.03 (0.36)^Ab^	2.96 (1.43)^Aa^
48 h	0.48 (0.01)^Ba^	0.46 (0.01)^Ba^	0.46 (0.00)^Ba^	0.47 (0.00)^Ba^	0.47 (0.01)^Ba^	4.67 (0.88)^Ab^	6.28 (3.16)^Ab^	8.27 (0.10)^Aab^	5.88(0.36)^Ab^	10.08 (1.84)^Aa^
Succinic acid										
24 h	14.72 (2.07)^Aa^	11.07 (2.61)^Aab^	10.81 (0.74)^Aab^	10.00 (0.01)^Ab^	12.15 (2.83)^Aab^	4.50 (0.93)^Ba^	3.16 (0.08)^Ba^	1.84 (0.14)^Ba^	5.36 (1.30)^Aa^	3.32 (0.16)^Ba^
48 h	21.89 (2.09)^Aa^	20.31 (8.10)^Aa^	20.14 (0.27)^Aa^	20.71 (1.08)^Aa^	15.67 (4.03)^Aa^	5.12 (1.45)^Ba^	4.28 (1.07)^Ba^	3.77 (0.60)^Ba^	5.59 (0.55)^Ba^	5.56 (0.76)^Ba^
Lactic acid										
24 h	1.17 (0.43)^Ac^	8.35 (0.74)^Ab^	9.83 (0.59)^Ab^	9.48 (0.31)^Ab^	13.75 (3.46)^Aa^	0.24 (0.00)^Aa^	0.66 (0.13)^Ba^	0.60 (0.04)^Ba^	0.61 (0.27)^Ba^	0.60 (0.00)^Ba^
48 h	1.47 (0.60)^Ac^	15.78 (5.50)^Ab^	13.04 (0.44)^Ab^	13.64 (0.57)^Ab^	27.26 (0.27)^Aa^	0.48 (0.01)^Aa^	2.49 (1.48)^Ba^	1.08 (0.20)^Ba^	1.16 (0.23)^Ba^	1.99 (0.76)^Ba^
Formic acid										
24 h	1.55 (0.19)^Ab^	3.25 (0.39)^Aa^	1.36 (0.05)^Ab^	1.98 (0.52)^Ab^	1.90 (0.22)^Ab^	0.40 (0.12)^Ba^	0.34 (0.13)^Ba^	0.51 (0.04)^Aa^	0.79 (0.27)^Ba^	0.72 (0.00)^Ba^
48 h	2.68 (0.45)^Ab^	5.03 (0.08)^Aa^	2.85 (0.77)^Ab^	2.92 (0.65)^Ab^	4.39 (0.38)^Aa^	1.04 (0.31)^Ba^	1.06 (0.24)^Ba^	1.37 (0.20)^Aa^	1.68 (0.23)^Ba^	2.13 (0.78)^Ba^
Caproic acid										
24 h	0.24 (0.00)^Aa^	0.23 (0.01)^Aa^	0.23 (0.00)^Aa^	0.24 (0.00)^Aa^	0.23 (0.00)^Ba^	0.35 (0.07)^Ab^	0.57 (0.26)^Aab^	0.40 (0.01)^Ab^	0.36 (0.01)^Ab^	0.78 (0.25)^Aa^
48 h	0.50 (0.01)^Aa^	0.46 (0.01)^Aa^	0.46 (0.00)^Aa^	0.47 (0.00)^Ba^	0.47 (0.01)^Ba^	0.77 (0.25)^Ab^	1.06 (0.49)^Ab^	0.63 (0.28)^Ab^	1.22 (0.16)^Aab^	1.77 (0.31)^Aa^
*i*-Butyric acid										
24 h	0.30 (0.07)^Ba^	0.21 (0.01)^Ba^	0.38 (0.01)^Ba^	0.27 (0.03)^Aa^	0.22 (0.01)^Aa^	0.76 (0.04)^Aa^	0.77 (0.03)^Aa^	0.76 (0.05)^Ba^	0.54 (0.24)^Aa^	0.66 (0.33)^Aa^
48 h	0.62 (0.14)^Aa^	0.53 (0.07)^Aa^	0.60 (0.02)^Aa^	0.48 (0.01)^Aa^	0.46 (0.01)^Ba^	1.42 (0.07)^Aa^	1.44 (0.59)^Aa^	1.84 (0.17)^Aa^	1.43 (0.20)^Aa^	1.92 (0.25)^Aa^
*i*-Valeric acid										
24 h	0.29 (0.01)^Ba^	0.25 (0.01)^Ba^	0.24 (0.00)^Ba^	0.24 (0.00)^Aa^	0.23 (0.01)^Ba^	1.21 (0.18)^Aa^	1.03 (0.19)^Aa^	1.08 (0.09)^Aa^	0.58 (0.23)^Aa^	0.99 (0.56)^Aa^
48 h	0.52 (0.00)^Ba^	0.46 (0.07)^Ba^	0.47 (0.00)^Ba^	0.47 (0.00)^Ba^	0.46 (0.01)^Ba^	2.65 (0.16)^Aa^	2.22 (1.13)^Aa^	2.51 (0.32)^Aa^	2.04 (0.04)^Aa^	3.10 (0.46)^Aa^

Values are expressed as mean (standard error). ^A,B^ Different capital letters in a row indicate significant differences (*P* < 0.05) in the cumulative SCFA, BCFA, and secondary organic acids amounts between different microbiotas (FMLI × FMOI) with the same test compound. ^a,b^Different lowercase letters in a row indicate significant differences (*P* < 0.05) in the cumulative SCFA, BCFA, and secondary organic acids amounts between the different test compounds for the same microbiota. Control = SIEM + dialysate; FS-Pla = SIEM + fermented soy beverage without the probiotic strains or the ABP; FS-Pro = SIEM + fermented soy beverage with the probiotic strains but without the ABP; FS-Pre = SIEM + fermented soy beverage with the ABP but without the probiotic strains; FS-Syn = SIEM + fermented beverage soy with the probiotic strains and the ABP

The cumulative iso-valeric acid was higher (*P* < 0.05) in the FMOI than in the FMLI, but no difference between the FS treatments and the control was observed (Table 2).

### Microbial compositional changes

**Fig. 2:**
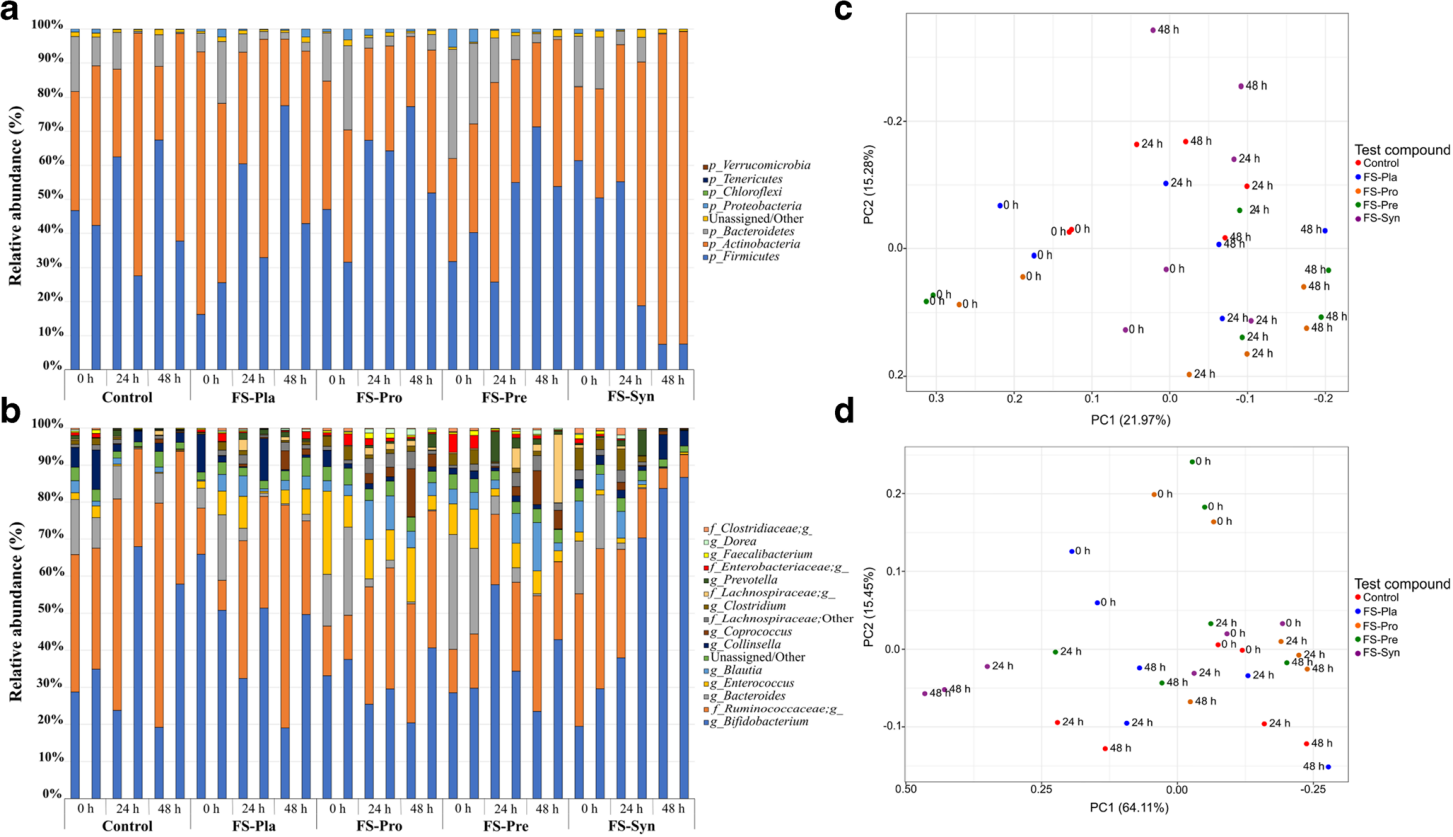
Relative abundance of the phyla (**a**) and genera (**b**) in the faecal microbiota from lean individuals (FMLI), for different test compounds in samples collected from the TIM-2 system at times *t* = 0 h (after the simulated lumen adaptation), *t* = 24 h, and *t* = 48 h. Unassigned and less abundant (< 0.5%) phyla and/or genera were grouped in “Unassigned/Others”. Principal Coordinate Analyses (PCoA) using unweighted UniFrac distance matrix of the FMLI (**c**). PCoA using weighted UniFrac distance matrix of the FMLI (**d**). The variance explained by the PCs is indicated in parentheses on the axes. Control = SIEM + dialysate solution; FS-Pla = SIEM + fermented soy beverage without the probiotic strains or the ABP; FS-Pro = SIEM + fermented soy beverage with the probiotic strains but without the ABP; FS-Pre = SIEM + fermented soy beverage with the ABP but without the probiotic strains; FS-Syn = SIEM + fermented beverage soy with the probiotic strains and the ABP

In the FMLI, *Firmicutes* increased in all treatments, except for FS-Syn (Fig. 2a), with an increase in unclassified genera of the family *Ruminococcaceae* and *Lachonospiraceae*, and the genera *Coprococcus* and *Dorea* (Fig. 2b). All treatments showed a decrease in the mean of relative abundance (RA%) of *Bacteroidetes* in the FMLI (7.43% for control; 9.53% for FS-Pla; 16.59% for FS-Pro; 26.01% for FS-Pre; and 14.76% for FS-Syn), representing mainly by the genus *Bacteroides*, which showed a decrease in the mean RA% of 26.72% for FS-Pre. A decrease in the *Actinobacteria* phylum was observed for beverages FS-Pla and FS-Pro, despite the presence of *B. longum* BB-46 in FS-Pro. The main changes were observed for FS-Pla, which showed a decrease of 29.81% in mean RA% of this phylum and a decrease in the genus *Bifidobacterium* (24.01%). On the other hand, FS-Pre and FS-Syn showed an increase in *Actinobacteria* in the FMLI, also represented mainly by an increase in *Bifidobacterium*, especially for FS-Syn, in which an increase of the mean RA% of 60.66% of this genus was observed. We observed a similarity between samples from *t* = 0 h for both matrices, as expected (Fig. 2c, d, and Supplementary information, Fig. S5), and this grouping was different to the other times of fermentation (*t* = 24 h and *t* = 48 h grouped) (PERMANOVA; unweighted: *P* = 0.001; weighted: *P* = 0.049). Furthermore, *t* = 48 h was significantly different from the other time points, but only for the unweighted UniFrac (PERMANOVA; unweighted: *P* = 0.001; weighted: *P* = 0.194), indicating a higher effect on microbiota richness than evenness. We did not observe significant differences between the four FSs for both distance matrices for the FMLI (PERMANOVA; unweighted: *P* = 0.347; weighted: *P* = 0.07).

**Fig. 3:**
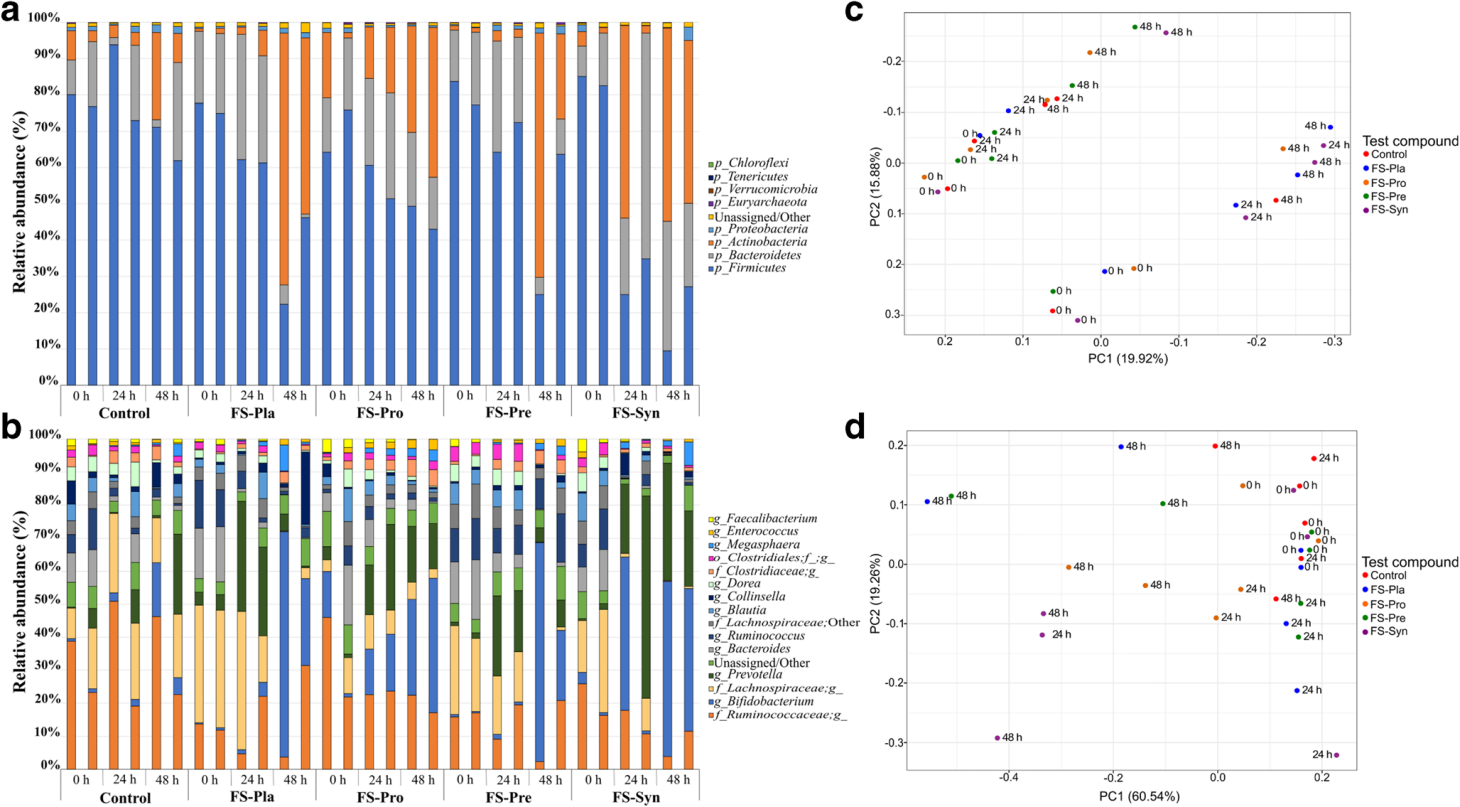
Relative abundance of the phyla (**a**) and genera (**b**) in the faecal microbiota from obese individuals (FMOI), for different test compounds in samples collected from the TIM-2 system at times *t* = 0 h (after the simulated lumen adaptation), *t* = 24 h, and *t* = 48 h. Unassigned and less abundant (< 0.5%) phyla and/or genera were grouped in “Unassigned/Others”. Principal Coordinate Analyses (PCoA) using unweighted UniFrac distance matrix of the FMOI (**c**). PCoA using weighted UniFrac distance matrix of the FMOI (**d**). The variance explained by the PCs is indicated in parentheses on the axes. Control = SIEM + dialysate solution; FS-Pla = SIEM + fermented soy beverage without the probiotic strains or the ABP; FS-Pro = SIEM + fermented soy beverage with the probiotic strains but without the ABP; FS-Pre = SIEM + fermented soy beverage with the ABP but without the probiotic strains; FS-Syn = SIEM + fermented beverage soy with the probiotic strains and the ABP

FMOI showed increases in the RA% of *Actinobacteria* during fermentation of all treatments (Fig. 3a) but mainly for the treatments with FS, in which an increase of 57.85%, 25.49%, 44.16%, and 46.32% in the mean RA% was observed, respectively, for FS-Pla, FS-Pro, FS-Pre, and FS-Syn, mainly due to an increase in the genera *Bifidobacterium* and *Collinsella* (Fig. 3b). Decreases in the *Firmicutes* phylum in the FMOI were observed for all experimental treatments (Fig. 3a), with decreases in the RA% of unclassified genera of the families *Ruminococcaceae* and *Lachonospiraceae* (Fig. 3b). The *Bacteroidetes* phyla decreased in FS-Pla and FS-Pre in the FMOI, as also observed for the FMLI, due to a decrease in the *Bacteroides* genus RA%. However, the reductions in the *Bacteroidetes* phyla were not observed for the control, FS-Pro, and FS-Syn treatments, due to increases in *Prevotella*, which was the fourth most abundant genus in the FMOI. Additionally, an increase (*P* < 0.05) in the *Prevotella/Bacteroides* ratio (from 0.11 at 0 h to 261.17 at 48 h) was observed for FS-Syn after 48 h of fermentation of the FMOI (Supplementary information, Table S5). Using unweighted (Fig. 3c and Supplementary information, Fig. S6A) and weighted UniFrac (Fig. 3d and Supplementary information Fig. S6B) distance matrices, significant differences were observed between samples in time *t* = 0 h and *t* = 48 h (*t* = 0 h: PERMANOVA; unweighted: *P* = 0.001; weighted: *P* = 0.001; *t* = 48 h: PERMANOVA; unweighted: *P* = 0.002; weighted: *P* = 0.001). However, as for the FMLI, in terms of the four FS treatments, no differences were observed for the FMOI (PERMANOVA; unweighted: *P* = 0.128; weighted: *P* = 0.414).

**Fig. 4:**
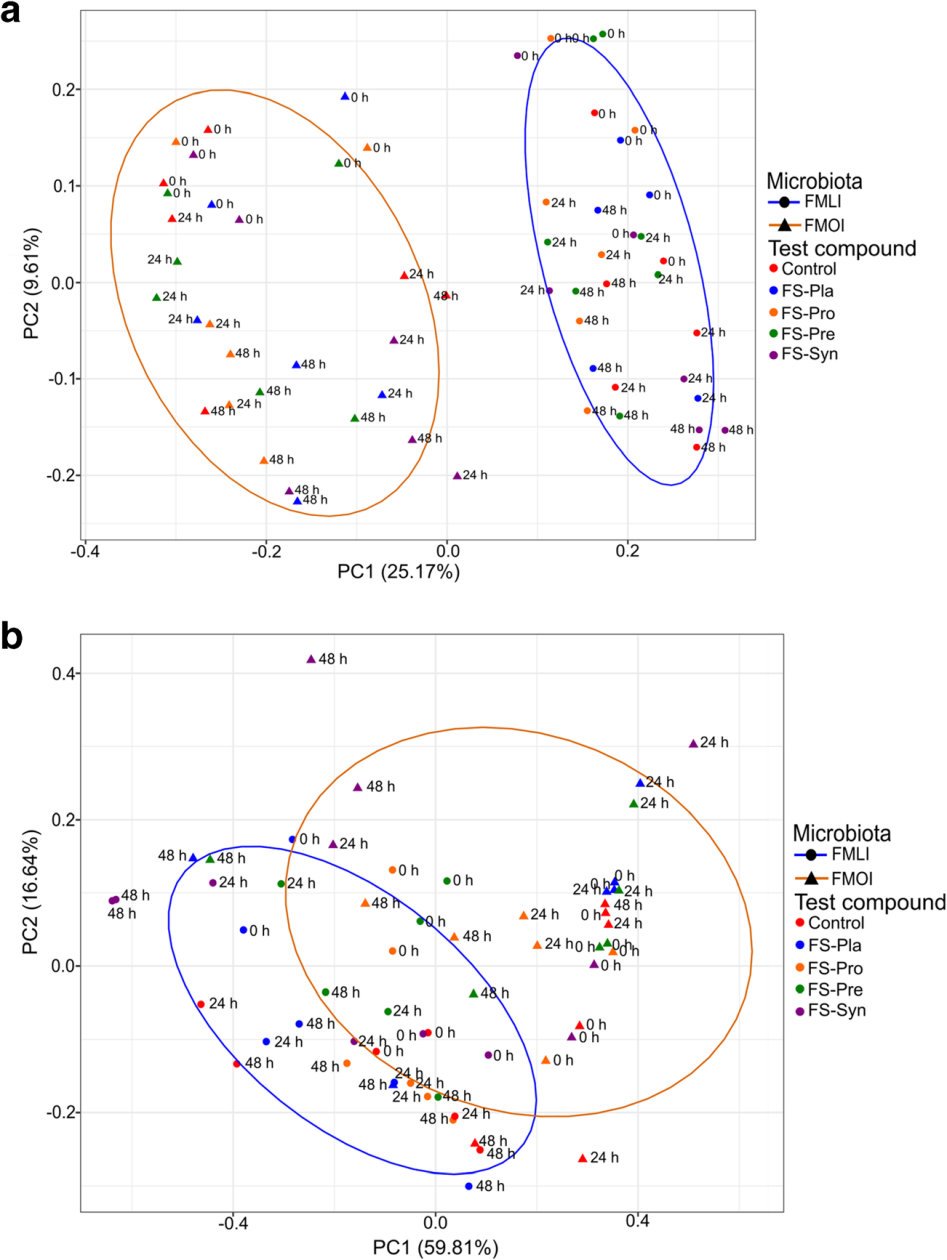
Principal Coordinate Analyses (PCoA) of lean vs. obese faecal microbiota using unweighted UniFrac (**a**) and weighted UniFrac (**b**) distance matrices. The variance explained by the PCs is indicated in parentheses on the axes. Control = SIEM + dialysate solution; FS-Pla = SIEM + fermented soy beverage without the probiotic strains or the ABP; FS-Pro = SIEM + fermented soy beverage with the probiotic strains but without the ABP; FS-Pre = SIEM + fermented soy beverage with the ABP but without the probiotic strains; FS-Syn = SIEM + fermented beverage soy with the probiotic strains and the ABP

When comparing changes of the FMLI and FMOI together, the unweighted UniFrac PCoA plots (Fig. 4a and Supplementary information, Fig. S7A) show a different microbiota community composition from the FMLI and FMOI (PERMANOVA; unweighted: *P* = 0.001; weighted: *P* = 0.001), even after the different fermentation conditions. This indicates that the composition of the two microbiotas was different, as could be observed from the relative abundance of genera. The difference between the FMLI and FMOI in the weighted UniFrac PCoA (Fig. 4b and Supplementary information, Fig. S7B) was less clear than for the unweighted but was still significant.

### Dynamics of total bacteria, *Bifidobacterium*, *Lactobacillus*, *L. acidophilus*, *B. longum*, and *S. thermophilus* in TIM-2 samples assessed by PMA-qPCR

**Fig. 5:**
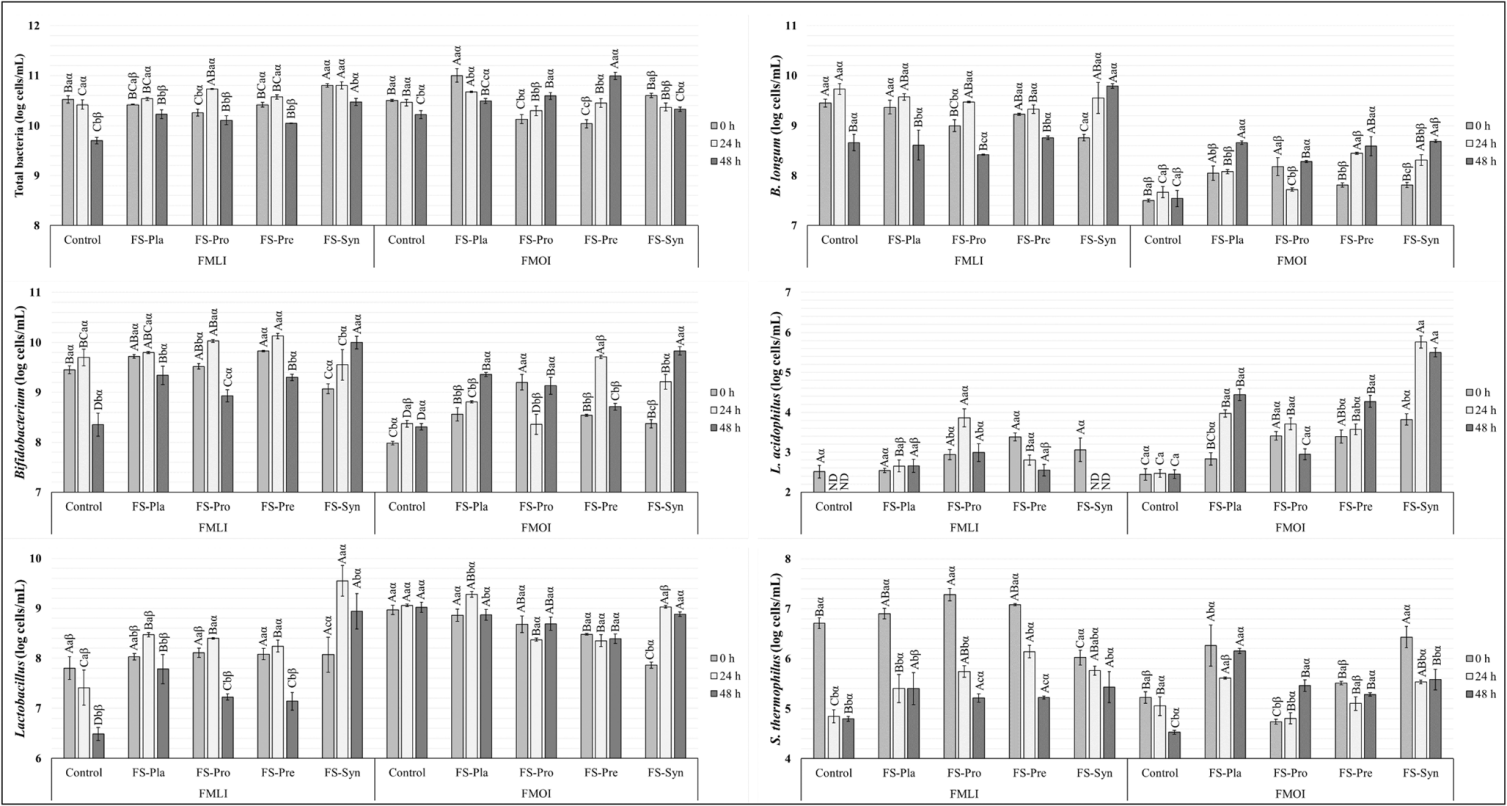
The populations of total bacteria, *Bifidobacterium* spp. and *Lactobacillus* spp., and the specific species *L. acidophilus*, *B. longum*, and *S. thermophilus*, obtained by PMA-qPCR for different test compounds in samples collected at times *t* = 0 h (after the simulated lumen adaptation), *t* = 24 h, and *t* = 48 h. ^A–D^Different superscript capital letters for each group and/or species of microorganism indicate significant differences (*P* < 0.05) between the different meals for the same microbiota and at the same time. ^a–c^Different superscript lowercase letters for each group and/or species of microorganism indicate significant differences (*P* < 0.05) between different times for the microbiotas with the same meal. ^α,β^Distinct superscript Greek letters for each group and/or species of microorganism indicate significant differences (*P* < 0.05) between different microbiotas for the same meal and the same time. ND = not determined, values bellow the detection limit (< 1.7 log cells/mL for *L. acidophilus*). Control = SIEM + dialysate solution; FS-Pla = SIEM + fermented soy beverage without the probiotic strains or the ABP; FS-Pro = SIEM + fermented soy beverage with the probiotic strains but without the ABP; FS-Pre = SIEM + fermented soy beverage with the ABP but without the probiotic strains; FS-Syn = SIEM + fermented beverage soy with the probiotic strains and the ABP. Values show mean (standard error) of two TIM-2 runs (log cells/mL) as calculated from Ct values

The changes in log cells per millilitre were assessed by PMA-qPCR for total bacteria and specific populations of the genera *Lactobacillus* and *Bifidobacterium*, and for the species *L. acidophilus*, *B. longum*, and *S. thermophilus.* Total bacterial, *Bifidobacterium*, and *Lactobacillus *populations showed differences (*P* < 0.05) for treatment and intervention time after fermentation for both microbiotas. The two specific genera were also different between the two microbiotas studied (*P* < 0.05) (Fig. 5). An increase (*P* < 0.05) in the *Bifidobacterium* and *Lactobacillus* populations was observed in both microbiotas when formulation FS-Syn was fed during 48 h of fermentation. However, this was not observed for FS-Pro and FS-Pre, both of which resulted in only maintaining these populations or even decreasing the populations of both genera, despite the addition of both genera in the case of FS-Pro. Thus, the increase in populations of bifidobacteria and lactobacilli occurred only when both factors (probiotics and ABP) were combined in an FS.

At 0 h, the *B. longum* populations were lower (*P* < 0.001) for the FMOI than for the FMLI. After 24 h of fermentation in the presence of the pre-digested FS, *B. longum* populations in the FMOI still remained lower (*P* < 0.001) than those observed for the FMLI. However, after 48 h, an increase (*P* < 0.05) up to 0.88 log cells/mL in the presence of FS-Pla, FS-Pre, and FS-Syn was observed for the FMOI, while a decrease (*P* < 0.05) in the *B. longum* populations in the FMLI was observed for all FSs and the control treatment, except for FS-Syn, which showed an increase up to 1 log cells/mL between 0 and 48 h. During fermentation with the different experimental treatments, *L. acidophilus* was not detected (< 1.7 log cells/mL) after 24 h in the FMLI for the control treatment and the FS-Syn, and no differences (*P* < 0.05) were observed in *L. acidophilus* populations for the other FSs between 0 and 48 h of fermentation for the FMLI. For the FMOI, an increase (*P* < 0.05) in *L. acidophilus* populations up to 1.7 log cells/mL was observed after 48 h of fermentation with FS-Pla, FS-Pre, and FS-Syn. A decrease (*P* < 0.05) in the *S. thermophilus* populations around 2 log cells/mL was observed for the FMLI, independently of the treatment provided. Also, a decrease (*P* < 0.05) in the *S. thermophilus* populations was observed for the FMOI for the control treatments and the FS-Syn.

## Discussion

A considerable amount of non-digested carbohydrates, proteins, and peptides from the diet reaches the colon, where they act as the main source of energy for the colonic microbiota. Consequently, they modify the gut microbiota composition and the gene expression encoding proteins and enzymes of metabolic pathways (Morrison and Preston 2016). The production of beneficial metabolites like SCFA are the primary end-products, resulting primarily from fermentation of these non-digested carbohydrates by the gut microbiota (Chambers et al. 2015; Morrison and Preston 2016; Murugesan et al. 2018). Among the SCFA produced from dietary fibre fermentation by the colonic microbiota, acetate prevails in relation to propionate and butyrate (Chambers et al. 2015), corroborating with the results here observed in FMLI and FMOI. A high production of SCFA by the gut microbiota is a positive feature that might prevent obesity through increased energy expenditure, production of the anorexic hormone, and appetite regulation (Canfora et al. 2019). This was observed for FMLI after fermentation in the presence of all FS. Regarding FMOI, the production of SCFA only increased for FS-Syn. Acetate was the most abundant SFCA observed in both microbiotas studied. If higher concentrations of acetate reach the brain, they might cross the blood-brain barrier and be absorbed predominantly by the hypothalamus. This absorption might promote an anorectic signal, mediating appetite suppression (Chambers et al. 2015). Butyrate plays an important role in the gene expression of epithelial and adipocyte cells (Chambers et al. 2015). This SCFA acts as an inhibitor of histone deacetylase, which regulates gene expression (Murugesan et al. 2018). Acetate and propionate induced adipogenicity by the FFA2 receptor (Byrne et al. 2015). Regarding lactate, high contents of this organic acid do not normally accumulate in the colon of healthy adult humans since these compounds may serve as substrates for other bacteria in the production of propionate and butyrate (Slavin 2013). Some strains belonging to the *Roseburia* genus and certain *Ruminococcaceae*, like *Faecalibacterium prausnitzii*, as well as *Lachnospiraceae*, present in the FMOI, are able to use acetate and lactate to produce butyrate (Kettle et al. 2015). However, no significant correlation between these SCFA and these OTUs was observed (data not shown), suggesting that these genera have different metabolic activities under the different treatments tested.

The branched-chain fatty acids (BCFA), *i*-butyrate and *i*-valerate, are produced by the gut microbiota in lower amounts than the SCFA (Rios-Covian et al. 2020) and are derived from protein and peptide fermentation (Canfora et al. 2019). These BCFA and other proteolytic metabolites like ammonia are generally considered to be harmful to the colon epithelium (Aguirre et al. 2016; Sáyago-Ayerdi et al. 2020). Although our previous study showed that FS is a good source of protein (containing > 17 g / 100 g of dry matter) (Vieira et al. 2019), no significant differences in the production of BCFA were observed between the FS and the control treatments. These results corroborate what was observed in a randomized clinical study that reported the absence of any significant correlation between protein intake and the production of BCFA (Rios-Covian et al. 2020). A previous study using the TIM-2 *in vitro* model showed that upon cassava bagasse feeding, the obese microbiota produced more BCFA than the lean microbiota (Souza et al. 2014), therefore similar to that which was obtained for *i*-valeric acid in the FMOI.

An increase in the abundance of the *Actinobacteria* members, particularly *Bifidobacterium* spp., has been considered beneficial in the human gut microbiota, due to correlations between low abundance of *Bifidobacterium* and obesity (Angelakis et al. 2012; Klancic and Reimer 2020; Million et al. 2013). As was shown here for FS-Syn, in a study in the SHIME® *in vitro* system, *B. longum* BB-46 showed positive effects on the lean gut microbiota when combined with ABP. This was due to a reduction in ammonium and an increase in SCFA concentration, as well as a reduction in the *Clostridium* spp. populations (Bianchi et al. 2019). Additionally, in the present study, FS-Syn showed an increase in the *Bifidobactereaceae* family in the FMLI, together with a decrease in the *Ruminococcaceae*, *Lachnospiraceae*, and *Bacteroidaceae* families (Supplementary information, Fig. S8). An increase in the *Bifidobacterium* populations in the faecal material was also reported during *B. animalis* subsp. *lactis* GCL2505 intervention in randomized clinical studies (Anzawa et al. 2019; Ishizuka et al. 2012). However, Ishizuka and colleagues did not observe changes in the *B. lactis* populations (Ishizuka et al. 2012), which contrasts with the present study, while Anzawa et al. (2019) showed an increase in the *B. animalis*, which was higher than what was observed for *B. longum* in FS-Syn in our study.

The increase in levels of *Prevotella* has been associated with high consumption of fibre in the diet, while the increase in the levels of *Bacteroides* was associated with high intake of fat and proteins (Wu et al. 2011). Although there is no consensus about health benefits of *Prevotella* due to the high genetic diversity within the genus (Precup and Vodnar 2019), studies show that the low abundance of *Prevotella* and low *Prevotella/Bacteroides* ratios have been correlated with obesity (Klinder et al. 2016; Kong et al. 2014). On the other hand, increases in *Prevotella* abundance and *Prevotella/Bacteroides* ratios were correlated with the improvement of glucose metabolism by promotion of glycogen storage in the liver after the intake of barley kernels (Kovatcheva-Datchary et al. 2015) and the reduction of serum triglyceride levels, as well as cardiovascular disease risk, after the consumption of β-glucan from barley (Wang et al. 2016).

Corroborating what was observed here, high abundance of *Lactobacillus* in obese individuals was reported (Crovesy et al. 2020). Cao et al. (2019) showed a correlation of lactobacilli with weight gain and induced obesity. Nonetheless, the obesogenic effect of the *Lactobacillus* genus is strain-specific (Crovesy et al. 2020; Drissi et al. 2014). In this sense, *Lactobacillus* strains with metabolic mechanisms for enhanced glycolysis and defences against oxidative stress might present an associated weight protection (Drissi et al. 2014). Although the species *L. acidophilus* has also been considered a possible obesogenic *Lactobacillus* (Drissi et al. 2014), some authors have reported that different strains of *L. acidophilus* showed advantageous effects on obesity and other associated diseases. For instance, Li et al. (2016) reported the anti-obesity effect of *L. acidophilus* AD031 on mice fed a high-fat diet (HFD) for 8 weeks by inducing a significantly lower food effect ratio (body weight gain/gram of food intake), on top of a significant decrease in serum triglyceride levels. The authors also reported inhibited serum activities of aspartate and alanine transaminase, as well as decreased lipid deposition in the liver, compared to the HFD group. Besides, increases in *Lactobacillus* abundance showed a potential for reducing blood glucose levels in obese people after intervention with a synbiotic food supplement containing four *Bifidobacterium* species and a *L. acidophilus* strain combined with galactooligosaccharides (GOS) (Sergeev et al. 2020).

In conclusion, in the present study, we have identified that FSs with probiotics and/or prebiotics resulted in different effects on the FMLI and FMOI. The synbiotic FS formulation (FS-Syn) increased the cumulative production of acetic, lactic, and formic acids for FMLI, and of valeric and caproic acids for FMOI after 48 h of fermentation in the TIM-2 *in vitro* model. An increase in the RA% of *Bifidobacterium* in the FMLI occurred for FS-Syn while all FS improve the RA% of this genus for FMOI. An increased population of *Lactobacillus* spp. was detected in both microbiota for FS-Syn, as well as a maintenance or an increase in the *L. acidophilus* populations in the FMOI, although *L. acidophilus* was not detected in FMLI. Regarding the FMLI and FMOI composition, a significant difference in the unweighted and weighted UniFrac was shown in both microbiotas between time 0 and 48 h of colonic fermentation in the presence of the different FSs. When comparing the changes of FMLI *vs.* FMOI, a shift of the FMOI profile into the FMLI profile space was observed over time as assessed by the weighted UniFrac. This indicated a change of the FMOI due to the fermentation of FSs, perhaps towards a healthier composition, with stimulation of beneficial microorganisms like *Bifidobacterium* and *Prevotella* and a decrease in the population members of the *Clostridiaceae* family. Moreover, the FSs supplemented with ABP and probiotic strains (*L. acidophilus* LA-5 and *B. longum* BB-46) may be used as a potential synbiotic food due to the bifidogenic effect observed in both microbiotas, also reducing the environmental impact caused by the current disposal of this fruit by-product. Although the results shown in the present study are promising, clinical trials are required to confirm the health benefits observed for the FSs studied.

## Supplementary information

ESM 1 (PDF 3736 kb)

## Data Availability

The datasets are available from the corresponding author on reasonable request. Raw sequences have been deposited in the European Nucleotide Archive under submission number PRJEB40878: (https://www.ebi.ac.uk/ena/browser/view/PRJEB40878).
