# Supplementary material for: Impact of a fermented soy beverage supplemented with acerola by-product on the gut microbiota from lean and obese subjects using an in vitro model of the human colon
Source: Appl Microbiol Biotechnol. 2021 May 3;105(9):3771–85. doi: 10.1007/s00253-021-11252-8 (PMC8102275; doi:10.1007/s00253-021-11252-8)
Supplement: Supplementary file 1 — (PDF 3736 kb) [file 253_2021_11252_MOESM1_ESM.pdf]

Applied Microbiology & Biotechnology

Impact of a fermented soy beverage supplemented with acerola by-product on the gut microbiota from lean and obese subjects using an *in vitro* model of the human colon

Antonio Diogo Silva Vieira[a,b], Carlota Bussolo de Souza[c], Marina Padilha[a,b], Erwin Gerard Zoetendal[d], Hauke Smidt[d], Susana Marta Isay Saad[a,b], and Koen Venema[c,*]

[a]*Department of Biochemical and Pharmaceutical Technology, School of Pharmaceutical Sciences, University of São Paulo, Av. Professor Lineu Prestes, 580, São Paulo, SP 05508-000, Brazil*

[b]*Food Research Center FoRC, University of São Paulo, Av. Professor Lineu Prestes, 580, São Paulo, SP 05508-000, Brazil*

[c]*Centre for Healthy Eating & Food Innovation, Maastricht University – Campus Venlo, St. Jansweg 20, 5928 RC Venlo, The Netherlands*

[d]*Laboratory of Microbiology, Wageningen University & Research, Stippeneng 4, 6708 WE, Wageningen, The Netherlands*

*Corresponding author e-mail address: k.venema@maastrichtuniversity.nl

Supplementary information

Supplementary Information Text S1 (Pre-digestion details)

FS were submitted to a pre-digestion in 3 steps (gastric phase, enteric phase I, and enteric II phase) (Buriti et al. 2010). Gastric enzymes (gastric pepsin and lipase with final concentration 3 g/L and 0.9 mg/L, respectively, both from Sigma-Aldrich, St. Louis, USA) were added to samples of 25 g of each beverage or dialysate solution, with pH set to 2.0-2.2, using 1 M HCl (Sigma-Aldrich, Munich, Germany). The mixture was incubated in a water bath (Julabo®, Seelbach, Germany) at 37 ºC for 2 h with constant agitation of approximate 150 rpm. Next, the pH was set to 4.5-4.7 through an alkaline solution of pH 12 (NaOH [6 g/L] and NaH_2PO_4 [10.8 g/L], Sigma-Aldrich). Bile (porcine bile, Sigma-Aldrich) and pancreatin (porcine pancreatin, Pancrex powder, Zoetis Belgium SA, Zaventem, Belgium) were added to a final concentration of 10 g/L and of 1 g/L, respectively. Incubation proceeded at 37 ºC for 2 h more to simulate the enteric phase I. Afterwards, the pH was set to 5.5-5.9, using the alkaline solution described above, containing bile and pancreatin maintained at concentrations of, respectively, 10 g/L and 1 g/L. A new incubation took place for another 2 h at 37 ºC for enteric II phase simulation.

Supplementary Information Text S2 (Microbiota analysis description)

Barcoded amplicons from the V3-V4 region of 16S rRNA genes were generated using a 2-step PCR. An amount of 10-25 ng genomic DNA was used as template for the first PCR with a total volume of 50 µl using the 341F (5'-CCTACGGGNGGCWGCAG-3') and the 785R (5'-GACTACHVGGGTATCTAATCC-3') primers appended with Illumina adaptor sequences. PCR products were purified, and the size of the PCR products were checked on a Fragment analyser (Advanced Analytical) and quantified by fluorometric analysis. Purified PCR products were used for the 2nd PCR in combination with sample-specific barcoded primers (Nextera XT index kit, Illumina). Subsequently, PCR products were purified, checked on a Fragment analyser (Advanced Analytical) and quantified, followed by multiplexing, clustering, and sequencing on an Illumina MiSeq with the paired-end (2x) 300 bp protocol and indexing. The sequencing run was analysed with the Illumina

CASAVA pipeline (v1.8.3) with demultiplexing based on sample-specific barcodes. The raw sequencing data produced was processed removing the sequence reads of too low quality (only "passing filter" reads were selected) and discarding reads containing adaptor sequences or PhiX control with an in-house filtering protocol. A quality assessment on the remaining reads was performed using the FASTQC quality control tool version 0.10.0. Quality trimming was applied based on Phred quality scores.

Supplementary information Text S3 (Bioinformatics description)

In order to ensure that comparable regions of the 16S rRNA gene were analysed across all reads, sequences that started before the 2.5-percentile or ended after the 97.5-percentile in the alignment were filtered. Potentially chimeric sequences were removed. Sequences were aligned and clustered into operational taxonomic units (OTUs; defined by 97% similarity). A biom table file was generated which was subsequently employed for diversity analysis. Rarefaction curves were computed with the "alpha_rarefaction.py", using Simpson metric and a rarefaction depth value of 1225 sequences. Principal Coordinate Analysis (PCoA) plots were obtained using the "beta_diversity_through_plots.py" command, selecting the weighted and unweighted UniFrac as desired metric to generate the distance matrix (Caporaso et al. 2010; Pruesse et al. 2007).

(A)

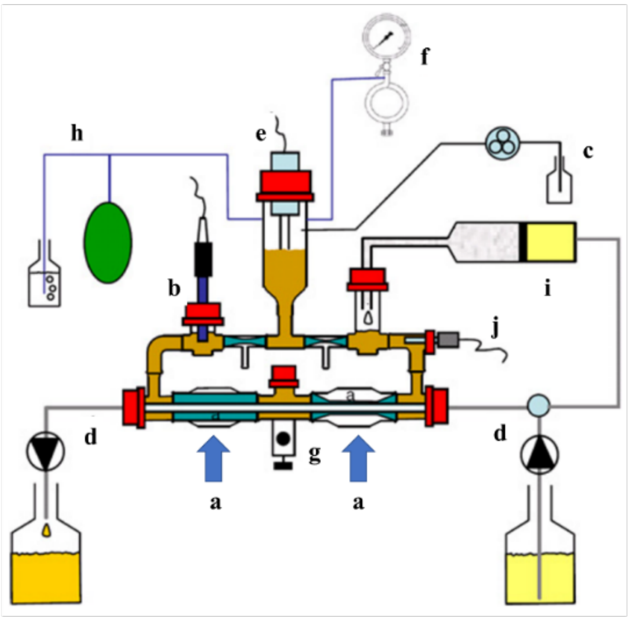

(B)

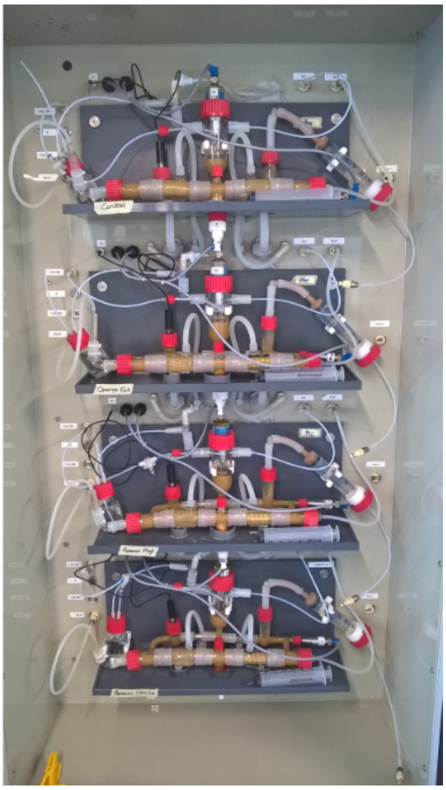

Figure S1. (A) Schematic representation of one unit of the large intestinal model (TIM-2 system) from Aguirre et al. (2014) and Minekus et al. (1999). (a) peristaltic compartments containing faecal material; (b) pH electrode; (c) alkali pump; (d) dialysis liquid circuit with hollow fibre membrane; (e) level sensor; (f) N_2 gas inlet; (g) sampling port; (h) gas outlet; (i) “ileal efflux” container (with SIEM); (j) temperature sensor. (B) Photograph of the cabinet with the 4 units of TIM-2.

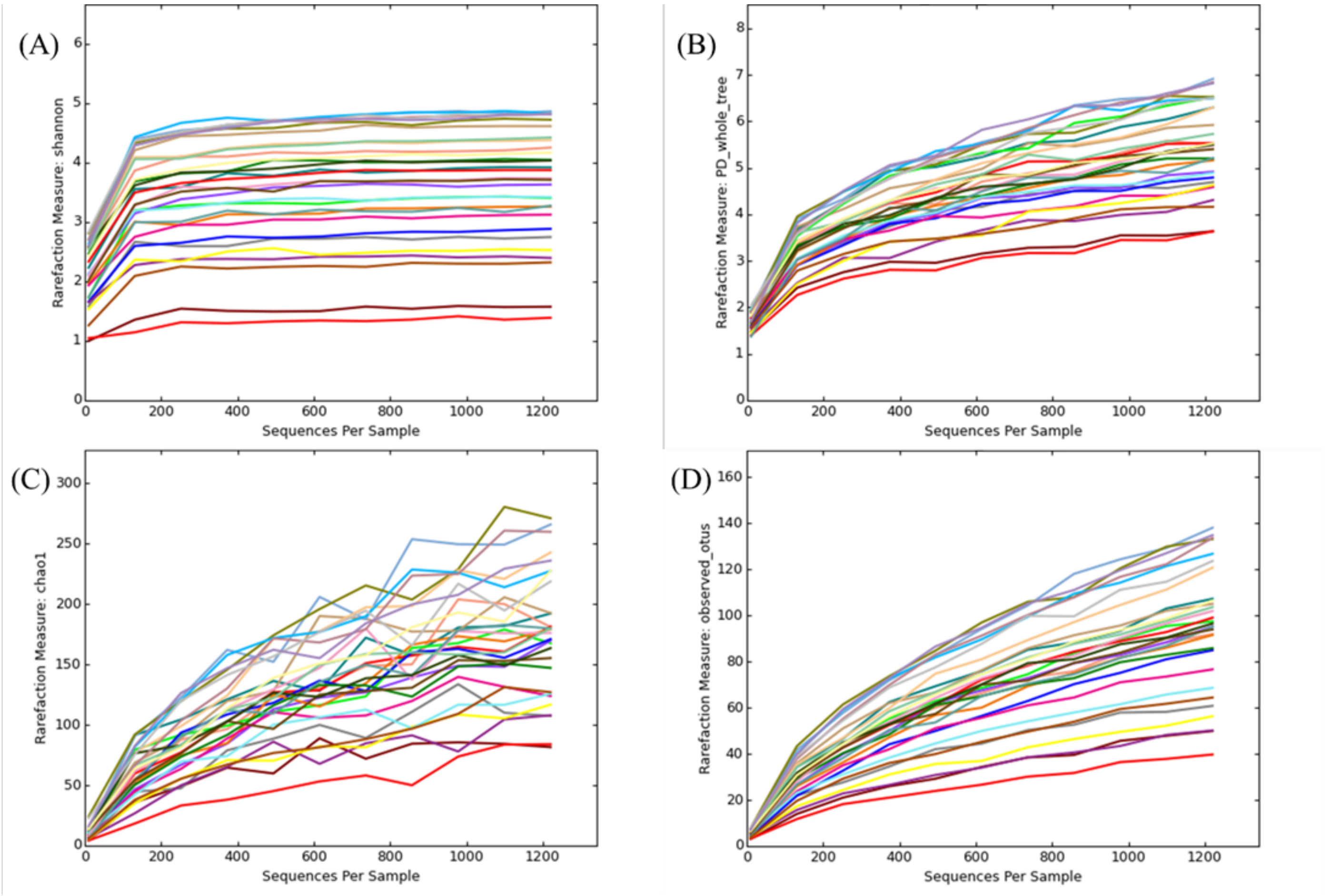

Figure 2S. Rarefaction curves of the α-diversity [(A) Shannon index, (B) PD whole tree, (C) Chao 1, and (D) Observed OTUs] observed for the FMLI obtained from TIM-2 trial using Simpson metric and a rarefaction depth value of 1225 sequences.

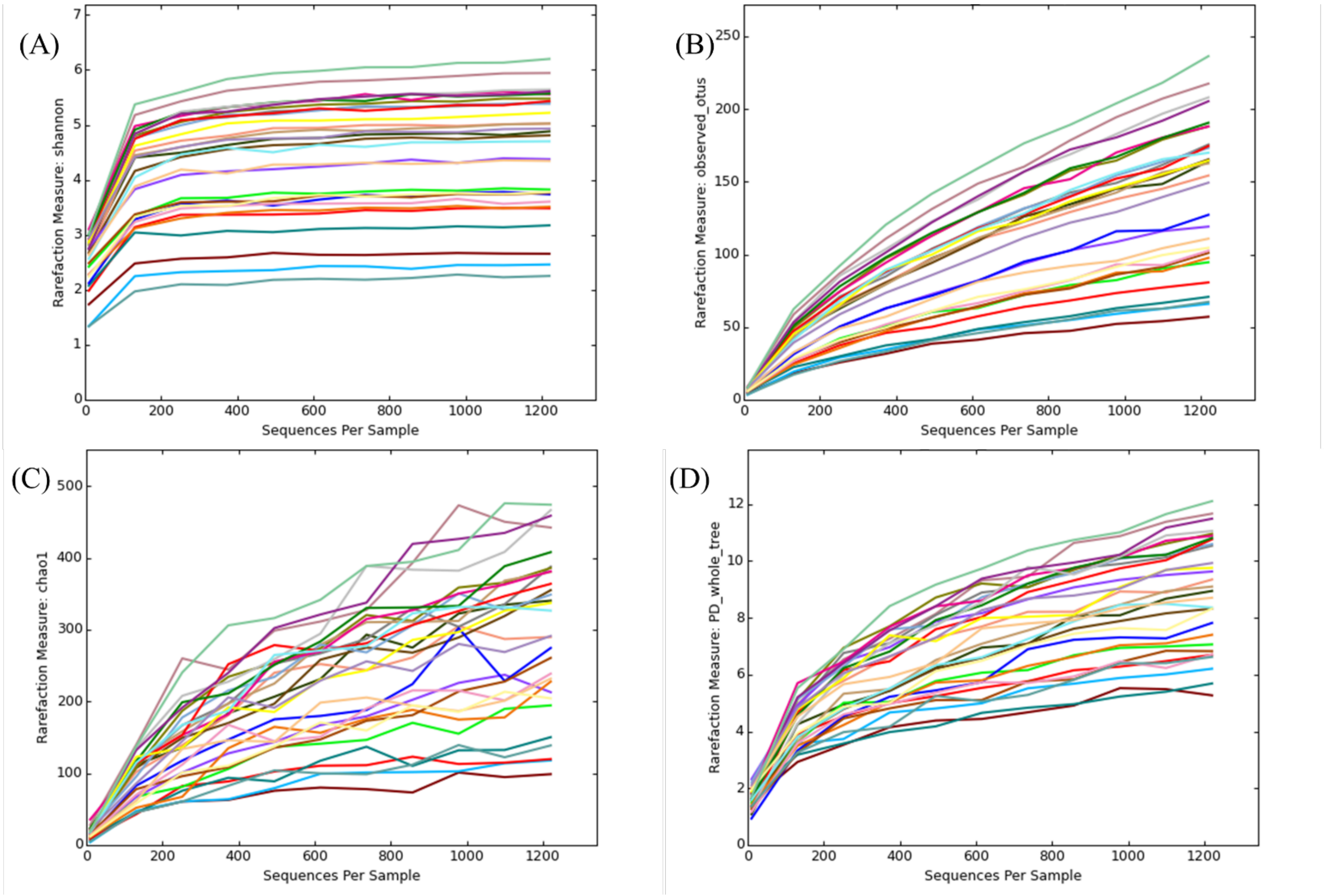

Figure 3S. Rarefaction curves of the α-diversity [(A) Shannon index, (B) PD whole tree, (C) Chao 1, and (D) Observed OTUs] observed for the FMOI obtained from TIM-2 trial using Simpson metric and a rarefaction depth value of 1225 sequences.

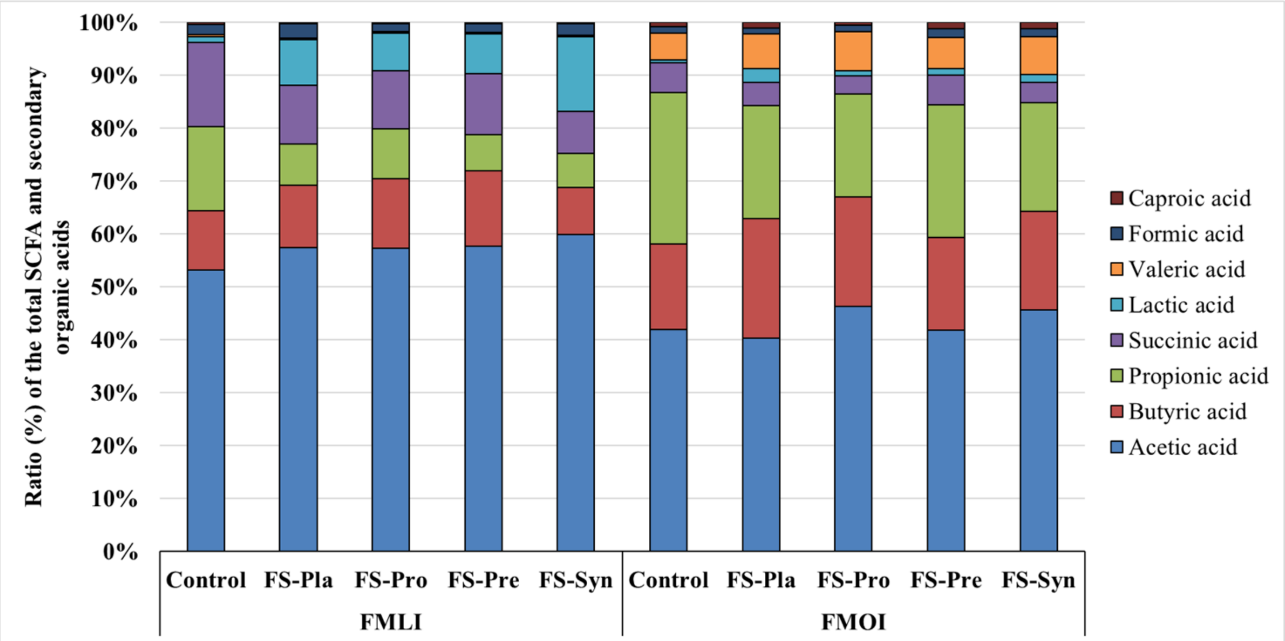

Figure S4. Short-chain fatty acids and secondary organic acids as ratios (%) from the different test compounds at 48 h for the faecal microbiota from lean individual (FMLI) and faecal microbiota from obese individuals (FMOI) tested. The simulated lumen were fed the following media: Control = SIEM + dialysate solution; FS-Pla- = SIEM + fermented soy beverage without the probiotic strains or the acerola by-product (ABP); FS-Pro = SIEM + fermented soy beverage with the probiotic strains but without the ABP; FS-Pre = SIEM + fermented soy beverage with the ABP but without the probiotic strains; FS-Syn = SIEM + fermented beverage soy with the probiotic strains and the ABP.

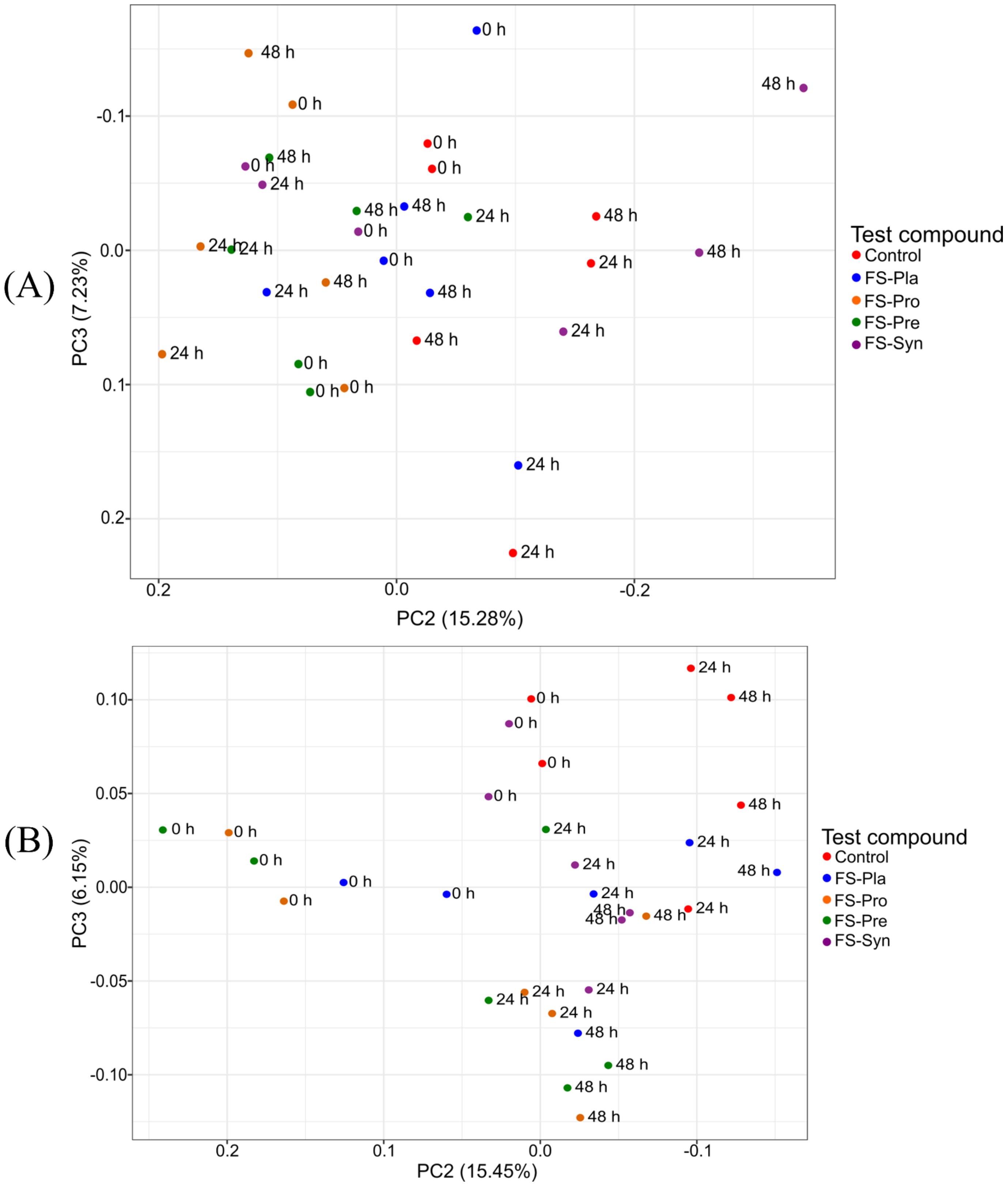

Figure 5S. (A) Principal Coordinate Analyses (PCoA, PC2 and PC3) using unweighted UniFrac distance matrix of the FMLI. (B) PCoA (PC2 and PC3) using weighted UniFrac distance matrix of the FMLI. The variance explained by the PCs is indicated in parentheses on the axes. Control = SIEM + dialysate solution; FS-Pla = SIEM + fermented soy beverage without the probiotic strains or the ABP; FS-Pro = SIEM + fermented soy beverage with the probiotic strains but without the ABP; FS-Pre = SIEM + fermented soy beverage with the ABP but without the probiotic strains; FS-Syn = SIEM + fermented beverage soy with the probiotic strains and the ABP.

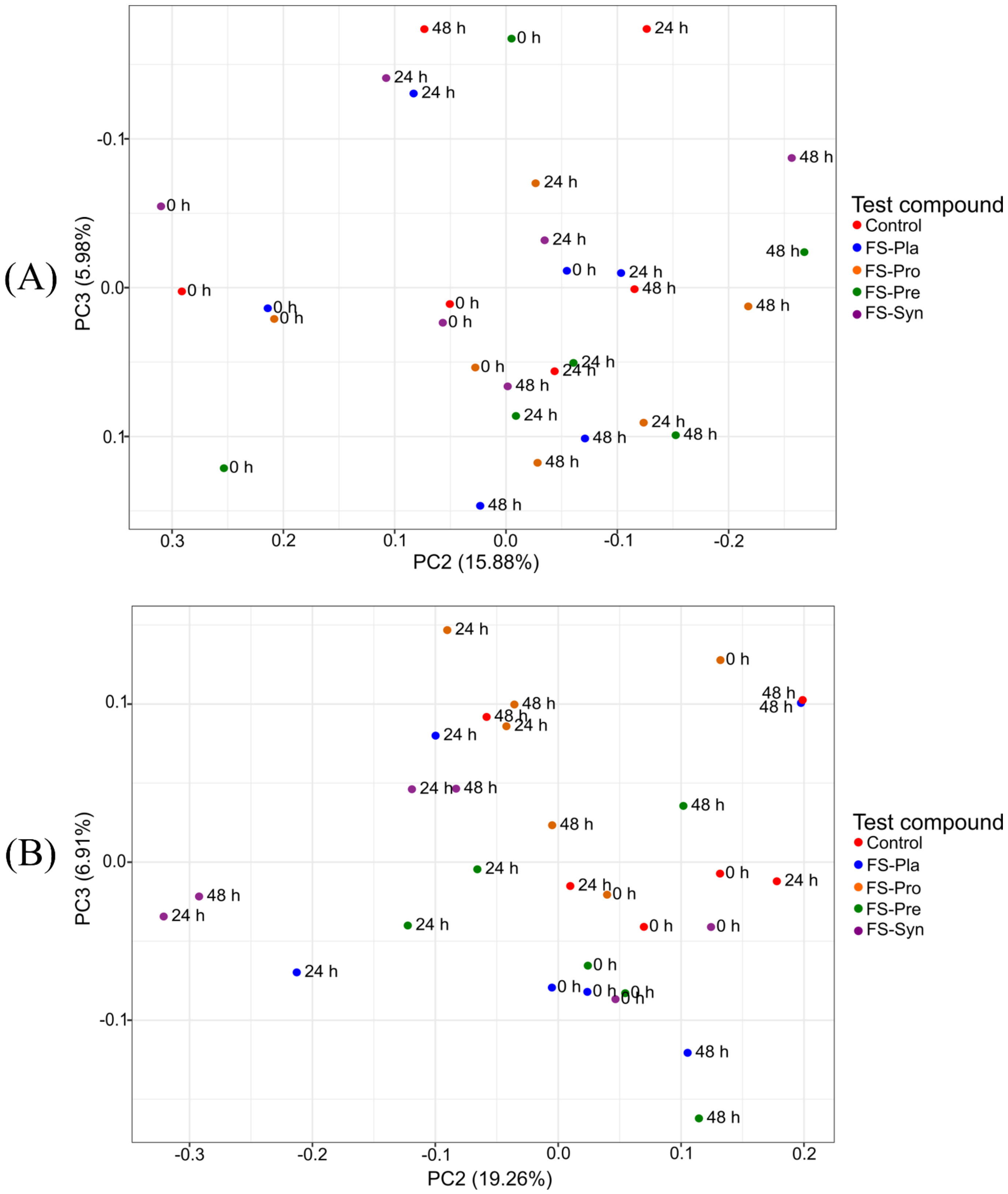

Figure 6S. (A) Principal Coordinate Analyses (PCoA, PC2 and PC3) using unweighted UniFrac distance matrix of the FMOI. (B) PCoA (PC2 and PC3) using weighted UniFrac distance matrix of the FMOI. The variance explained by the PCs is indicated in parentheses on the axes. Control = SIEM + dialysate solution; FS-Pla = SIEM + fermented soy beverage without the probiotic strains or the ABP; FS-Pro = SIEM + fermented soy beverage with the probiotic strains but without the ABP; FS-Pre = SIEM + fermented soy beverage with the ABP but without the probiotic strains; FS-Syn = SIEM + fermented beverage soy with the probiotic strains and the ABP.

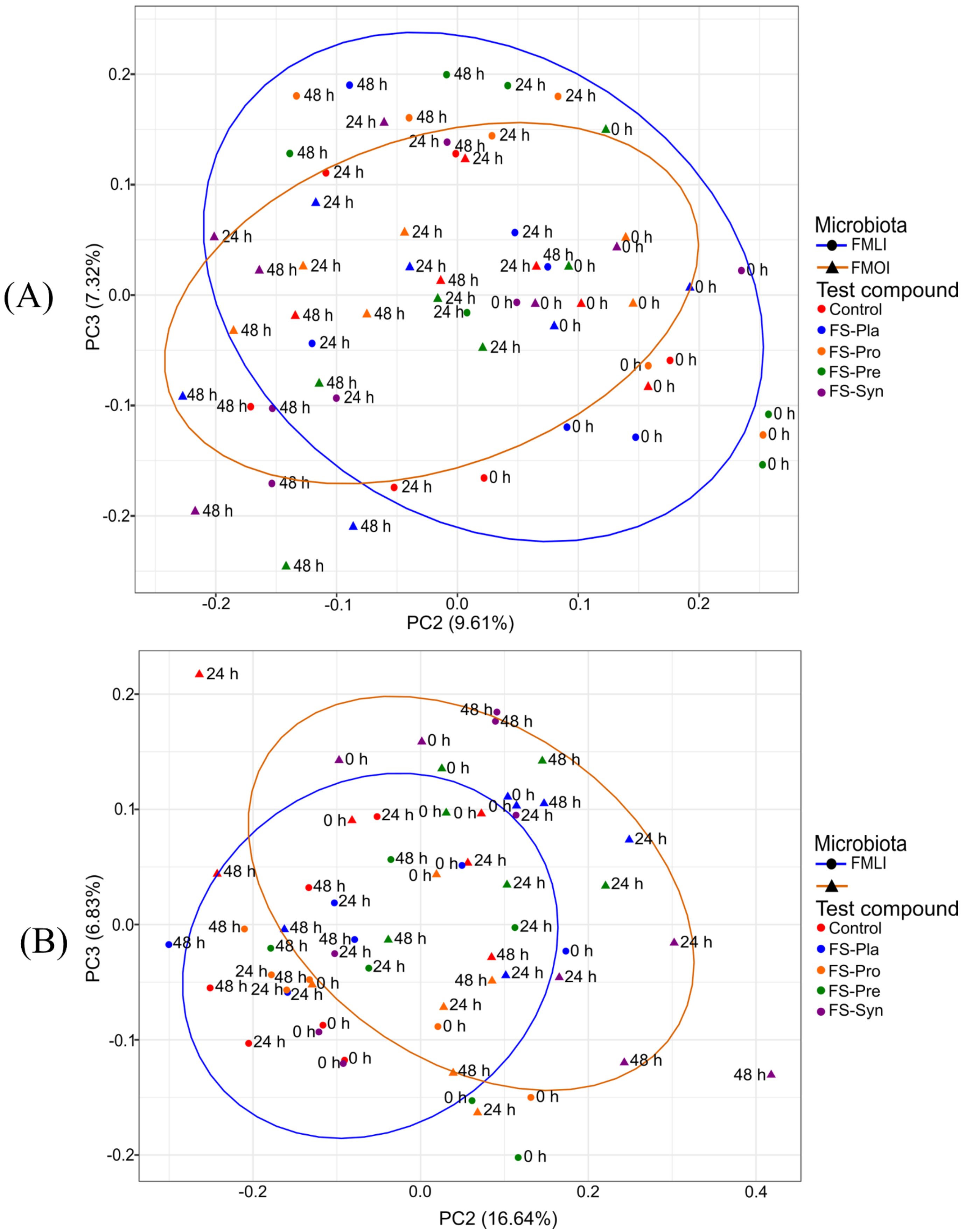

Figure 7S. (A) Principal Coordinate Analyses (PCoA, PC2 and PC3) using unweighted UniFrac distance matrix of the FMLI vs. FMOI. (B) PCoA (PC2 and PC3) using weighted UniFrac distance matrix of the FMLI vs. FMOI. The variance explained by the PCs is indicated in parentheses on the axes. Control = SIEM + dialysate solution; FS-Pla = SIEM + fermented soy beverage without the probiotic strains or the ABP; FS-Pro = SIEM + fermented soy beverage with the probiotic strains but without the ABP; FS-Pre = SIEM + fermented soy beverage with the ABP but without the probiotic strains; FS-Syn = SIEM + fermented beverage soy with the probiotic strains and the ABP.

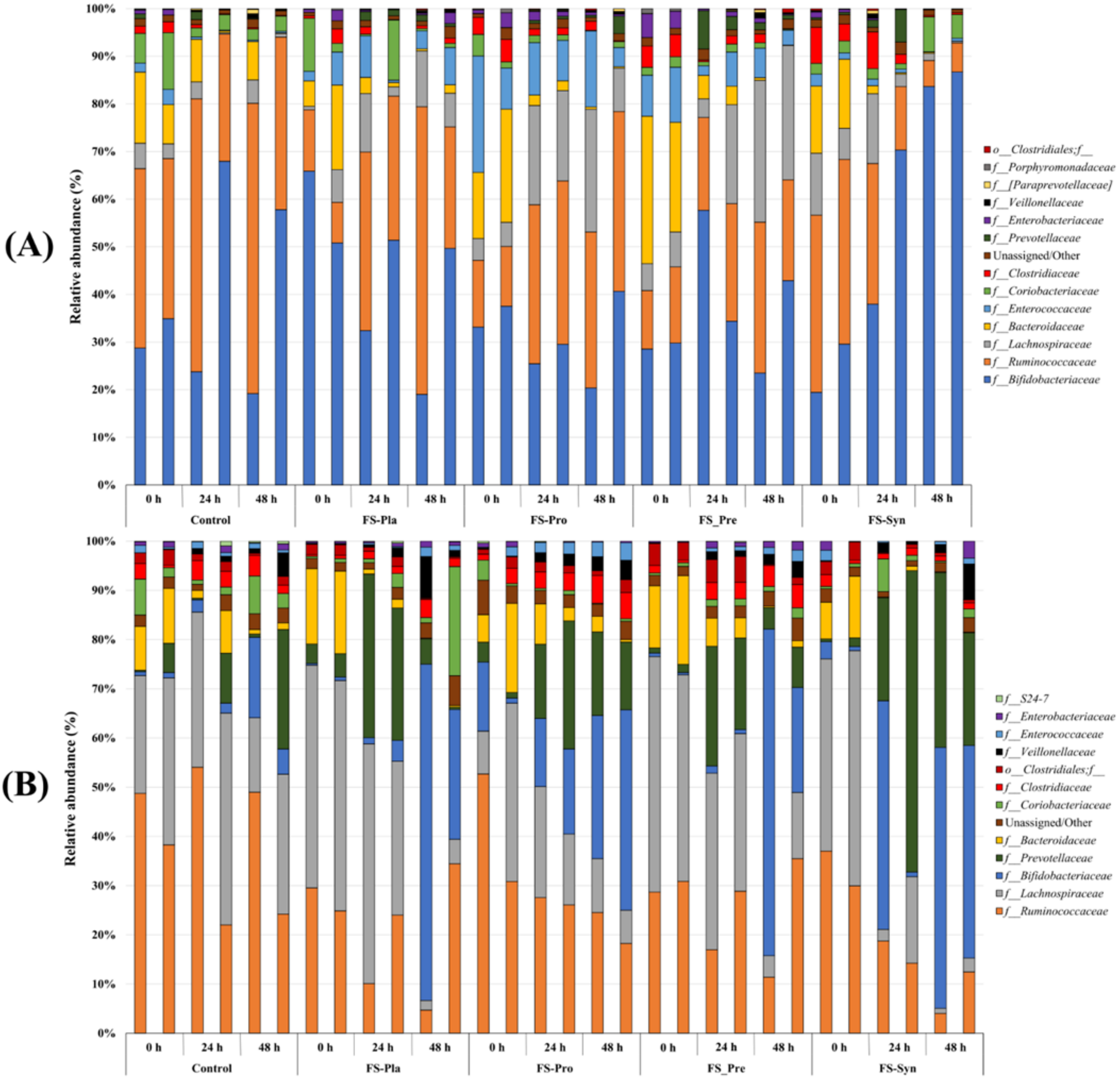

Figure S8. Relative abundance of the families in the faecal microbiota from lean individuals (A) and in faecal microbiota from obese individuals (B) from TIM-2 system, for the fermented soy beverages and the control at sample collection times 0 h (after the microbiota stabilization), 24 h and 48 h. Unassigned and less abundant (< 0.5%) families were grouped in "Unassigned/Others". Control = SIEM + dialysate solution; FS-Pla = SIEM + fermented soy beverage without the probiotic strains or the ABP; FS-Pro = SIEM + fermented soy beverage with the probiotic strains but without the ABP; FS-Pre = SIEM + fermented soy beverage with the ABP but without the probiotic strains; FS-Syn = SIEM + fermented beverage soy with the probiotic strains and the ABP.

Table S1

Composition of dialysate solution (Dial)

Ingredient	Concentration
K_2HPO_4 (Sigma-Aldrich, Munich, Germany)	1.90 $g.L^{-1}$
NaCl (Sigma-Aldrich)	4.50 $g.L^{-1}$
$FeSO_47H_2O$ (Sigma-Aldrich)	5.00 $mg.L^{-1}$
$MgSO_4$ H_2O (Sigma-Aldrich)	0.50 $g.L^{-1}$
$CaCl_2$ $2H_2O$ (Sigma-Aldrich)	0.45 $g.L^{-1}$
Ox-bile (Merck, Buchs, Switzerland)	50.0 $mg.L^{-1}$
L-Cysteine (Sigma-Aldrich)	0.40 g.L-1
Vitamin mixture* (Tritium Microbiology, Eindhoven, The Netherlands)	1.00 $mL.L^{-1}$

*Vitamin mixture containing (per litter): 1 mg menadione, 2 mg D-biotin, 0.5 mg vitamin B12, 10 mg pantothenate, 5 mg nicotinamide,5 mg p-aminobenzoic acid and 4 mg thiamine.

Table S2.

Composition of *Standard Ileal Efflux Media* (SIEM)

**Ingredient**	**Concentration ($g.L^{-1}$ or $mL.L^{-1}$)**
Pectin from citrus (Sigma-Aldrich)	12
Xylan (Sigma-Aldrich),	12
Arabinogalactan (Avebe, Veendam, The Netherlands)	12
Soluble starch (Fisher Scientific, Loughborough, Leicestershire, UK)	100
TBCO x6.25[1] (Tritium Microbiology)	25
Magnesium sulphate hexahydrate solution (5%, w/v, Sigma-Aldrich)	2
L-cysteine HCL solution (2%, w/v, Sigma-Aldrich)	2
Electrolytes mix solution[2] (Tritium Microbiology)	4

[1] TBCO (270 g/L Tween 80, 375 g/L bacterial peptone, 375 g/L casein, 6.25 g/L ox-bile)

[2] Electrolytes mix solution (25 g/L $K_2HPO_4.3H_2O$, 45 g/L NaCl, 4.5 g/L $CaCl_2.2H_2O$, 0.05 g/L $FeSO_4.7H_2O$, 0.1 g/L)

Table S3

qPCR reactions information

Target	Standard cultures	Primer and probes	Sequence 5'-3'	Concentration (nM)	Master Mix	Reference
Total bacteria	*Escherichia coli* DH5α [1]	F_Bact 1369	CGGTGAATACGTTCCCGG	200	1 x TaqMan Universal[3]	Furet et al. (2009)
		R_Prok1492	TACGGCTACCTTGTTACGACTT	200		
		P_TM1389F	6FAM-CTTGTACACACCGCCCGTC-TAMRA	250		
Bifidobacterium spp.	*Bifidobacterium longum* BB-46 [2]	F_Bifid 09c	CGGGTGAGTAATGCGTGACC	200	1 x TaqMan Universal[3]	Furet et al. (2009)
		R_Bifid 06	TGATAGGACGCGACCCCA	200		
		P_Bifid	6FAM-CTCCTGGAAACGGGTG-TAMRA	250		
Lactobacillus spp.	*Lactobacillus acidophilus* LA-5 [2]	Lac-F	AGCAGTAGGGAATCTTCCA	500	1x Power Sybr Green PCR[3]	Rinttilä et al. (2004)
		Lac-R	CAC CGC TAC ACA TGG AG	500		
Bifidobacterium longum group	*Bifidobacterium longum* BB-46 [2]	Bif_L_F	TTCCAGTTGATCGCATGGTCTTCT	200	1x Power Sybr Green PCR[3]	Adapted from Gueimonde et al. (2007)
		Bif_L_R	GGCTACCCGTCGAAGCCACG	200		
Lactobacillus acidophilus	*Lactobacillus acidophilus* LA-5 [2]	Acidfor	AGCGAGCTGAACCAACAGAT	200	1x Power Sybr Green PCR[3]	Tabasco et al. (2007)
		Acidrev	AGGCCGTTACCCTACCAACT	200		
Streptococcus thermophilus	*Streptococcus thermophilus* TH-4[2]	Strep_t_F	GTTCACACTGTGACGGTAGCTT	500	1x Power Sybr Green PCR[3]	Falentin et al. (2012)
		Strep_t_R	GAGCCACAGCCTTTAACTTCAGA	500		

[1]Thermo Fisher Scientific, Waltham, USA; [2]Christian Hansen, Hørsholm, Denmark; [3]Applied Biosystem, Foster City, USA

Table S4

Average (standard error) of α-diversity measures (Shanonn Index, PD_whole_tree, Chao 1, and Observed_OTUs) observed for faecal microbiota from lean individuals (FMLI) and faecal microbiota form obese individuals (FMOI), obtained from TIM-2 trials with the different test compounds tested.

		**FMLI**				**FMOI**			
**Test compounds**	**Time (h)**	**Shannon index**	**PD_whole_tree**	**Chao 1**	**Observed_OTUs**	**Shannon index**	**PD_whole_tree**	**Chao 1**	**Observed_OTUs**
**Control**	**0**	3.96 (0.02)$^{Ca\beta}$	5.366 (0.08)$^{BCa\beta}$	164.24 (7.87)$^{Ca\beta}$	92.40 (1.95)$^{Ba\beta}$	5.50 (0.11)$^{Aa\alpha}$	10.79 (0.02)$^{Aa\alpha}$	385.84 (13.62)$^{Aa\alpha}$	182.50 (2.60)$^{ABa\alpha}$
	**24**	2.64 (0.06)$^{Db\beta}$	4.548 (0.07)$^{Db\beta}$	139.44 (9.49)$^{Ca\beta}$	67.50 (4.08)$^{Db\beta}$	4.67 (0.44)$^{Bb\alpha}$	9.65 (0.22)$^{Bb\alpha}$	366.55 (24.07)$^{Aa\alpha}$	166.25 (9.08)$^{Bb\alpha}$
	**48**	2.89 (0.08)$^{Cb\beta}$	4.895 (0.08)$^{Bb\beta}$	147.99 (11.33)$^{Ca\beta}$	71.00 (4.16)$^{Cb\beta}$	4.36 (0.30)$^{Ab\alpha}$	8.58 (0.20)$^{Ac\alpha}$	282.45 (16.88)$^{Ab\alpha}$	131.05 (7.81)$^{Ac\alpha}$
**FS-Pla**	**0**	3.56 (0.04)$^{Da\beta}$	5.144 (0.10)$^{Cb\beta}$	116.55 (6.37)$^{Db\beta}$	81.25 (3.12)$^{Cb\beta}$	4.75 (0.12)$^{Ba\alpha}$	8.34 (0.02)$^{Ba\alpha}$	340.74 (11.31)$^{Ba\alpha}$	167.60 (1.54)$^{Ba\alpha}$
	**24**	3.24 (0.11)$^{Cb\beta}$	5.089 (0.10)$^{Cb\beta}$	171.87 (7.71)$^{BCa\beta}$	81.35 (4.81)$^{Cb\beta}$	4.51 (0.45)$^{Ba\alpha}$	8.62 (0.21)$^{Ca\alpha}$	313.20 (21.83)$^{Ba\alpha}$	139.00 (8.54)$^{Cb\alpha}$
	**48**	3.34 (0.02)$^{Bb\alpha}$	5.865 (0.17)$^{Aa\beta}$	180.42 (8.03)$^{Ba\alpha}$	96.25 (1.40)$^{Ba\alpha}$	3.04 (0.15)$^{Cb\alpha}$	6.84 (0.18)$^{Bb\alpha}$	166.68 (9.68)$^{Cb\alpha}$	80.95 (3.32)$^{Cc\beta}$
**FS-Pro**	**0**	4.34 (0.02)$^{Bb\beta}$	5.611 (0.07)$^{Bb\beta}$	250.93 (8.45)$^{Aa\beta}$	97.75 (1.69)$^{Bc\beta}$	5.61 (0.33)$^{Aa\alpha}$	10.73 (0.14)$^{Aa\alpha}$	381.85 (22.47)$^{ABa\alpha}$	195.25 (9.49)$^{Aa\alpha}$
	**24**	4.84 (0.02)$^{Aa\alpha}$	6.865 (0.05)$^{Aa\beta}$	235.18 (13.97)$^{Aa\beta}$	136.40 (1.02)$^{Aa\beta}$	5.16 (0.12)$^{Ab\alpha}$	10.26 (0.05)$^{ABa\alpha}$	319.97 (11.13)$^{Bb\alpha}$	161.25 (3.09)$^{Bb\alpha}$
	**48**	4.27 (0.03)$^{Ab\alpha}$	5.912 (0.11)$^{Ab\beta}$	199.41 (8.56)$^{ABb\alpha}$	113.60 (2.14)$^{Ab\alpha}$	4.06 (0.11)$^{Ac\alpha}$	8.52 (0.07)$^{Ab\alpha}$	218.02 (7.53)$^{Bc\alpha}$	107.65 (0.96)$^{Bc\alpha}$
**FS-Pre**	**0**	4.72 (0.03)$^{Aa\beta}$	6.212 (0.10)$^{Aa\beta}$	197.51 (20.07)$^{Ba\beta}$	114.30 (4.82)$^{Aa\beta}$	5.33 (0.26)$^{Aa\alpha}$	10.08 (0.07)$^{Ab\alpha}$	423.37 (18.37)^{Aa}	185.25 (5.54)$^{Aa\alpha}$
	**24**	3.92 (0.18)$^{Bc\beta}$	5.553 (0.23)$^{Bb\beta}$	198.60 (8.70)$^{Ba\beta}$	104.85 (6.59)$^{Ba\beta}$	5.52 (0.08)$^{Aa\alpha}$	10.92 (0.02)$^{Aa\alpha}$	383.01 (14.82)$^{Aa\alpha}$	188.00 (1.64)$^{Aa\alpha}$
	**48**	4.23 (0.14)$^{Ab\alpha}$	5.704 (0.19)$^{Ab\beta}$	226.18 (10.65)$^{Aa\alpha}$	110.95 (3.80)$^{Aa\alpha}$	3.42 (0.40)$^{Bb\beta}$	7.92 (0.22)$^{Ac\alpha}$	165.41 (12.16)$^{Cb\alpha}$	92.55 (6.16)$^{Bb\beta}$
**FS-Syn**	**0**	4.43 (0.09)$^{Ba\beta}$	6.168 (0.17)$^{Aa\beta}$	177.87 (6.21)$^{BCa\beta}$	115.05 (4.39)$^{Aa\beta}$	5.41 (0.34)$^{Aa\alpha}$	10.31 (0.12)$^{Aa\alpha}$	381.24 (14.96)$^{Aa\alpha}$	190.75 (6.17)$^{Aa\alpha}$
	**24**	3.12 (0.18)$^{Cb\beta}$	5.228 (0.25)$^{BCb\beta}$	145.28 (6.98)$^{Cb\beta}$	85.80 (4.98)$^{Cb\alpha}$	3.47 (0.18)$^{Cb\alpha}$	6.26 (0.07)$^{Db\alpha}$	205.49 (15.46)$^{Cb\alpha}$	85.90 (3.64)$^{Db\alpha}$
	**48**	1.48 (0.02)$^{Dc\beta}$	3.635 (0.03)$^{Cc\beta}$	80.78 (6.15)$^{Dc\alpha}$	44.80 (1.26)$^{Dc\beta}$	3.07 (0.20)$^{BCc\alpha}$	5.98 (0.10)$^{Cb\alpha}$	109.29 (3.06)$^{Dc\alpha}$	68.90 (2.75)$^{Cc\alpha}$

$^{A-D}$ Different capital letters in a column indicate significant differences (Kruskal-Wallis, $P<0.05$) between the different test compounds for a same simulated lumen and at the same time. $^{a-c}$ Different lowercase letters in a column indicate significant differences (Kruskal-Wallis, $P<0.05$) between different times for the simulated lumen with the same test compound. $^{\alpha,\beta}$ Distinct Greek letters in a column indicate significant differences (Mann-Whitney U, $P<0.05$) between different simulated lumen for the same test compound and the same time. *Test compounds fed to the simulated lumen were: Control = SIEM + dialysate solution; FS-Pla- = SIEM + fermented soy beverage without the probiotic strains or the ABP; FS-Pro = SIEM + fermented soy beverage with the probiotic strains but without the ABP; FS-Pre = SIEM + fermented soy beverage with the ABP but without the probiotic strains; FS-Syn = SIEM + fermented beverage soy with the probiotic strains and the ABP.

Table S5. Means of the ratio *Prevotella/Bacteroides* obtained from relative abundance obtained from TIM-2 trials with the control (dialysate solution) and the different fermented soy beverages.

Test compounds	FMLI		FMOI	
	Time			
	0 h	48 h	0h	48h
**Control**	0.04 (0.03)	0.26 (0.24)	0.28 (0.25)	9.18 (8.47)
**FS-Pla**	0.00 (0.00)	1.84 (1.49)	0.27 (0.01)	23.54 (21.96)
**FS-Pro**	0.00 (0.00)	7.50 (6.59)	0.39 (0.33)	17.06 (11.69)
**FS-Pre**	0.00 (0.00)	12.94 (10.06)	0.09 (0.01)	10.94 (4.56)
**FS-Syn**	0.03 (0.00)	0.50 (0.50)	0.11 (0.03)#	261.17 (29.83)#

Value showed as mean (standard error). FMLI – Faecal microbiota from lean individuals. FMOI – Faecal microbiota from obese individuals. # Significant difference (P<0.05) between 0 h and 48 h obtains from non-parametric Mann-Whitney U test. *Test compounds fed to the simulated lumen were: Control = SIEM + dialysate solution; FS-Pla- = SIEM + fermented soy beverage without the probiotic strains or the ABP; FS-Pro = SIEM + fermented soy beverage with the probiotic strains but without the ABP; FS-Pre = SIEM + fermented soy beverage with the ABP but without the probiotic strains; FS-Syn = SIEM + fermented beverage soy with the probiotic strains and the ABP.

References

Aguirre M, Jonkers DMAE, Troost FJ, Roeselers G, Venema K (2014) *In vitro* characterization of the impact of different substrates on metabolite production, energy extraction and composition of gut microbiota from lean and obese subjects. Plos One, 26:e113864. https://doi.org/10.1371/journal.pone.0113864.

Buriti FCA, Castro IA, Saad SMI (2010) Viability of *Lactobacillus acidophilus* in synbiotic guava mousses and its survival under *in vitro* simulated gastrointestinal conditions. Int J Food Microbiol 137:121-129. doi:10.1016/j.ijfoodmicro.2009.11.030.

Caporaso JG, Kuczynski J, Stombaugh J, Bittinger K, Bushman FD, Costello EK, Fierer N, Peña AG, Goodrich JK, Gordon JI, Huttley GA, Kelley ST, Kinghts D, Koenig JE, Ley RE, Lozupone, CA, McDonald D, Muegge BD, Pirrung M, Reeder J, Sevinsky JR, Turnbaugh PJ, Walters WA, Widmann JR, Yatsunenko T, Zaneveld J, Knight, R. (2010). QIIME allows analysis of high-throughput community sequencing data. Nat Methods 7(5):335-336. doi:10.1038/nmeth.f.303.

Falentin H, Henaff N, Bivic PL, Deutsch S-M, Parayre S, Richoux R, Sohier D, Thierry A, Lortal S, Postollec F (2012) Reverse transcription quantitative PCR revealed persistency of thermophilic lactis acid bacteria, metabolic activity until the end of the ripening of Emmental cheese. Food Microbiol 29:132-140. doi:10.1016/j.fm.2011.09.009.

Furet J-P, Firmesse O, Gourmelon M, Bridonneau C, Tap J, Mondot S, Doré J, Corthier G (2009). Comparative assessment of human and farm animal faecal microbiota using real-time quantitative PCR. FEMS Microbiol Ecol 68:351-362. https://doi.org/10.1111/j.1574-6941.2009.00671.x.

Gueimonde M, Debor L, Tölkkö S, Jokisalo E, Salminen S (2007) Quantitative assessment of faecal bifidobacteria populations by real-time PCR using lanthanide probes. J Appl Microbiol 102:1116-1122. doi:10.1111/j.1365-2672.2006.03145.x.

Minekus M, Smeets-Peeters M, Bernalier A, Marol-Bonnin S, Havenaar R, Marteau P, Alric M, Fonty G, Huis in't Veld JHJ (1999). A computer-controlled system to simulate conditions of the large intestine with peristaltic mixing, water absorption and absorption of fermentation products. Appl Microbiol Biotechnol, 53:108-114. DOI: 10.1007/s002530051622.

Pruesse E, Quast C, Knittel K, Fuchs BM, Lugwing W, Peplies J, Glöckner FO (2007) SILVA: a comprehensive online resource for quality checked and aligned ribosomal RNA sequence data compatible with ARB. Nucleic Acids Res, 35(21):7188-7196. doi:10.1093/nar/gkm864.

Rinttilä T, Kassinen A, Malinen E, Krogius L, Palva A (2004) Development of an extensive set of 16S rDNA-targeted primers for quantification of pathogenic and indigenous bacteria in faecal samples by real-time PCR. J Appl Microbiol 9:1166-1177. doi:10.1111/j.1365-2672.2004.02409.x.

Tabasco R, Paarup T, Janer C, Peláez C, Requena T (2007) Selective enumeration and identification of mixed cultures of *Streptococcus thermophilus, Lactobacillus delbrueckii* subsp. *bulgaricus, L. acidophilus, L. paracasei* subsp. *paracasei* and *Bifidobacterium lactis* in fermented milk. Int Dairy J 17:1107-1114. doi:10.1016/j.idairyj.2007.01.010.
